# Multi-Tasking and Choice of Training Data Influencing Parietal ERP Expression and Single-Trial Detection—Relevance for Neuroscience and Clinical Applications

**DOI:** 10.3389/fnins.2018.00188

**Published:** 2018-03-27

**Authors:** Elsa A. Kirchner, Su Kyoung Kim

**Affiliations:** ^1^Robotics Lab, University of Bremen, Bremen, Germany; ^2^Robotics Innovation Center, German Research Center for Artificial Intelligence (DFKI), Bremen, Germany

**Keywords:** P300, prospective positivity, single-trial classification, classifier transfer, human-machine interaction

## Abstract

Event-related potentials (ERPs) are often used in brain-computer interfaces (BCIs) for communication or system control for enhancing or regaining control for motor-disabled persons. Especially results from single-trial EEG classification approaches for BCIs support correlations between single-trial ERP detection performance and ERP expression. Hence, BCIs can be considered as a paradigm shift contributing to new methods with strong influence on both neuroscience and clinical applications. Here, we investigate the relevance of the choice of training data and classifier transfer for the interpretability of results from single-trial ERP detection. In our experiments, subjects performed a visual-motor oddball task with motor-task relevant infrequent (*targets*), motor-task irrelevant infrequent (*deviants*), and motor-task irrelevant frequent (*standards*) stimuli. Under *dual-task* condition, a secondary senso-motor task was performed, compared to the *simple-task* condition. For evaluation, average ERP analysis and single-trial detection analysis with different numbers of electrodes were performed. Further, classifier transfer was investigated between simple and dual task. Parietal positive ERPs evoked by *target* stimuli (but not by deviants) were expressed stronger under dual-task condition, which is discussed as an increase of task emphasis and brain processes involved in task coordination and change of task set. Highest classification performance was found for *targets* irrespective whether all 62, 6 or 2 parietal electrodes were used. Further, higher detection performance of *targets* compared to *standards* was achieved under *dual-task* compared to *simple-task* condition in case of training on data from 2 parietal electrodes corresponding to results of ERP average analysis. Classifier transfer between tasks improves classification performance in case that training took place on more varying examples (from dual task). In summary, we showed that P300 and overlaying parietal positive ERPs can successfully be detected while subjects are performing additional ongoing motor activity. This supports single-trial detection of ERPs evoked by target events to, e.g., infer a patient's attentional state during therapeutic intervention.

## 1. Introduction

In many brain-computer interface (BCI) applications (Vidal, 1973; Wolpaw et al., 2002) the detection of the well-known event-related potential (ERP) P300 is used for communication (Farwell and Donchin, 1988; Riccio et al., 2011) or control of computer programs and machines, including complex virtual environments such as a virtual apartment (Bayliss, 2003) or robots (Kim et al., 2014), which can be used with the goal of compensating for motor actions that a patient cannot carry out. In such settings, the attention of the user is clearly focused on the presented stimuli used for control purposes or the control task itself. On the other hand, in embedded Brain Reading (eBR) applications (Kirchner et al., 2010, 2013; Woehrle and Kirchner, 2014b,a; Kirchner et al., 2016) a P300 might be evoked by stimuli that are part of an human-machine interaction (Kirchner et al., 2016). For example, in our previous study (Kirchner et al., 2016), the strength of P300 expression evoked by stimuli that are inherently part of the interaction task can directly be used to estimate the workload of the user in, e.g., a teleoperation scenario. Thus, it was not necessary to add an extra task which has usually been performed to investigate the effect of different parameters on the workload such as in previous studies (Isreal et al., 1980; Allison and Polich, 2008; Käthner et al., 2014). This indicates that single-trial ERP classification can be used not only for control purposes in BCIs but also as a measure for the strength in expression of ERPs in single trial to infer e.g., on the workload of a user online. This is a good example that BCIs can contribute to a paradigm shift in EEG analysis.

BCIs have also been applied to detect motor-related brain activity in single trials for rehabilitation purposes (McFarland et al., 2010; Folgheraiter et al., 2012; Ramos-Murguialday et al., 2013; Donati et al., 2016; Osuagwu et al., 2016). As with the P300-based BCIs that are applied for control purposes, online estimation of the attentional state of a patient might also be of relevance in rehabilitation application. During a therapeutic intervention it might be useful to combine both the detection of activity evoked by target recognition as well as activity evoked by the planning of motor activity. For example, the task of a patient in a therapeutic intervention is to try to move the affected arm. This task can be commanded by an event in a serious game for rehabilitation or given by the therapist (Wöhrle et al., 2017). Based on the strength of P300 expression, the strain on the patient could be estimated and the game automatically be adapted. Hence, for motor rehabilitation applications, i.e., therapeutic interventions, it is very relevant to infer how well a patient is able to attend therapy to avoid to overstrain him or her and consequently get best results in therapy. Our earlier work showed that motor activity can be detected in conditions that are highly interactive, i.e., involving complex mental and motor activities of the human (Kirchner and Drechsler, 2013; Kirchner et al., 2013; Woehrle and Kirchner, 2014a) and not only under conditions in which a subject tries to mentally control a rehabilitation device. However, the influence of *multi-tasking* on ERP expressions that are related with target recognition has not been well investigated for multi-motor-tasking but often only for a combination of a mental, visual or audio task with a visual or visual-motor task (Isreal et al., 1980; Kramer et al., 1995; Käthner et al., 2014; Ke et al., 2016).

In the presented study, we developed a scenario which allows to investigate the effect of *multi-tasking* on ERP expression that is related with recognition of task-relevant information, which elicits a combination of P300 components. P300 is a well-known ERP evoked by infrequent stimuli which is in some way perceived and attended by subjects (Isreal et al., 1980; Polich, 2007). Depending on the application and relevance of the infrequent stimuli, different types of P300 components are expected to be evoked. The more *frontocentral P3a* is known to be evoked by deviant non-target events, the *parietocentral P3b* is evoked by the evaluation of stimuli that require an overt or covert response, and the *frontal novelty P3* mainly reflects involuntary attention shifts (Squires et al., 1975; Duncan-Johnson and Donchin, 1977; Verleger et al., 1994; Polich, 2007). In most eBR applications and many other BCI applications, a combination of P300 sub-components is likely evoked. For example, when teleoperating robots with an interface that gives additional feedback on the situation within the environment of the robot or on the situation of the robot itself (see for example Kirchner et al., 2016), information might be presented that has a task-relevance for the operator, i.e., the operator has to react on them. Other information might be infrequent but irrelevant for the behavior of the operator. A similar example can be given for rehabilitation applications. An instruction given by the therapist is usually task-relevant. Additional information, e.g., on performance of the patient that is infrequent and will be attended but is not directly task relevant can be provided by, e.g., a serious game or the therapist. Hence, in both types of application, it is expected that different P300 components can be evoked, i.e., P3a on task-irrelevant infrequent stimuli, P3b evoked by task-relevant infrequent stimuli and even frontal novelty P3 can be expected to be evoked by novel unexpected events. This is different to classical experimental settings under controlled conditions of, e.g., an oddball discrimination task (Picton, 1992), where only specific stimuli are presented that are expected to evoke specific ERPs, i.e., task-irrelevant infrequent stimuli will evoke a P3a and not a P3b. It is therefore of interest to investigate ERP activity under less controlled application-close conditions.

Most studies using multi-tasking paradigms which are related to target recognition have investigated P300 expression depending on different task conditions (e.g., different workload levels). Indeed, it is well known that P300 in amplitude is influenced by changes in workload and attention and the workload can affect different P300 components (see Kok, 2001; Miller et al., 2011). These findings are consistent with our previous findings that P300 amplitude was reduced by unattended target stimuli or in case of lacking resources to cognitively process target stimuli (Woehrle and Kirchner, 2014b,a; Kirchner et al., 2016). In Kirchner et al. (2016), a reduction in single-trial P300 classification (caused by a reduction in P300 amplitude) was used as an indicator that an operator of a robotic systems is overstrained in a complex interaction scenario. Furthermore, we observed that the operator missed task-relevant information (targets) in a very complex interaction scenario and in this case the missing of a P300 and thus the failure in detection of a target trial (i.e., correct classification of non-target) due to the missing of a P300 can be used as a sign of too much strain on the interacting subject or inattentiveness (Kirchner et al., 2016). However, in our previous studies, we did not compare the multi-motor-task condition in the eBR application with a simple-motor-task condition and similar studies investigating this are missing as per to our knowledge. Therefore, we developed a scenario which contains two conditions, i.e., a simple-task condition (visual-motor oddball) and a dual-task condition involving a visual-motor oddball and a competing complex motor behavior. In the developed scenario in the presented study, subjects were overtrained in both tasks to avoid too much strain under the dual-task condition which may result in a strong reduction of P300 amplitude (cf. a complex interaction scenario in which an operator of a robotic systems is overstrained (Kirchner et al., 2016).

While the effect of workload on P300 expression has been investigated very well under dual-task condition contributing strongly to the early roots of a framework of multiple resource theory (Kahneman, 1973; Isreal et al., 1980; Parasuraman and Davies, 1984; Wickens, 1992, 2008; Polich, 2007), the effect of competing motor tasks was rarely investigated. One study from literature that comes close to our setting was published by Fowler (1994). In this study, both, the competing motor tasks, i.e., flying and landing a virtual aircraft and responding to a secondary oddball task, requesting a motor response were combined. However, task-irrelevant infrequent stimuli (deviants) were not presented and investigated concurrently. Studies that make use of the oddball paradigm typically make use of either (1) task-irrelevant *frequent* (standards) and task-irrelevant *infrequent* stimuli (deviants) (see for example Kramer et al. (1995) making use of the irrelevant-probe technique Papanicolaou and Johnstone, 1984) or (2) task-*irrelevant* frequent (standards) and task-*relevant* infrequent stimuli (deviants) (Isreal et al., 1980; Kramer et al., 1983). While under such *separate* conditions a P3a is expected to be evoked under condition (1) by task-irrelevant infrequent stimuli, a P3b is expected to be evoked under condition (2) by task-relevant infrequent stimuli. To our knowledge there is no study combining (1) and (2) with *a complex sensor-motor task* as in the presented study under dual-task condition and no study with a visual-motor oddball as primary task.

In human-machine interactions that require multi-motor behavior of the human, the importance and effects of multi-tasking on the attention and recognition as well as differentiation between task-relevant and infrequent but task-irrelvant events are of high relevance. In our recent BR applications it was of relevance, whether task-relevant stimuli are recognized and cognitively processed. A failure in recognition can stem from mental overload, attentional shifts or cognitive failures in processing, e.g., failure in recognition of task relevance. All these conditions are relevant for different applications (e.g., teleoperation, rehabilitation, etc.). Based on the earlier mentioned expectations on involved brain processes and expected ERP activities, we focus our analysis on ERPs at parietal electrodes. Further, electrode reduction, which is considered for applicability in BCI applications, is also focused on parietal electrodes. Hence, in the presented study we investigate a physiologically supported electrode reduction schemata concentrating on parietal electrodes. Further, we investigate the effect of classifier transfer between conditions on single-trial ERP detection, since for BCI applications (including eBR applications) classifier training can take place on examples of classes that are similar to the one of interest. Classifier transfer is applied for different reasons: (1) not enough training data is available (Kirchner et al., 2013; Woehrle and Kirchner, 2014b,a), (2) training data is time-consuming to acquire (Kim and Kirchner, 2013, 2016; Kim et al., 2017), or (3), as presented in this study, classifier transfer promises a better distinguishability. The impact of such transfer approaches on the physiological interpretability is not well investigated and will therefore be investigated in the presented study. The question that comes along with classifier transfer is, whether the general distinguishability of classes or the commonality of patterns is more relevant for the outcome of single-trial classification and how this can be interpreted regarding underlying brain processes.

In summary, the main goal of this study is to answer the following questions:

1. Is the general observed pattern in averaged partietal ERP activity mirrored by single-trial classification performance as suggested by preliminary investigations in Kirchner et al. (2013)?
2. Are the strong parietal ERP expressions found in BR applications (Kirchner et al., 2013, 2016; Woehrle and Kirchner, 2014a) likely to result from overlaying parietal ERP activity?
3. What impact a knowledge-driven electrode reduction approach has on classification performance and physiological interpretability of results? Can the obtained classification performance be expected from knowledge on evoked ERP components?
4. What influence a classifier transfer (e.g., see Kirchner et al., 2013; Kim and Kirchner, 2013, 2016; Kim et al., 2017) has on the interpretability of single-trial classification performance?
5. What is the relevance of the results obtained in the presented study for neuroscience methods as well as rehabilitation robotics?

To answer these questions the paper will be structured as follows. First we perform an average ERP analysis to investigate activity expected at parietal electrode sides. Next, we investigate separability of EEG activity in single trial under different conditions with respect to electrode selection by performing a single-trial analysis. This analysis should give an insight into the issues whether performance in single-trial classification correlates with the strength of expression of average ERP activity at parietal electrode sites. Especially, we investigate how strongly ERP activity at parietal electrodes can contribute to the classification performance. To this end, we perform a single-trial analysis with three binary classifications: task-relevant infrequent events (targets) vs. task-irrelevant frequent events (standards), task-irrelevant infrequent events (deviants) vs. task-irrelevant frequent events (standards), and task-relevant infrequent events (targets) vs. task-irrelevant infrequent events (deviants). Before these binary classifications we performed a multi-class classification to check distinguishability between the three classes (standards, targets, deviants). Additionally, we investigate the effect of classifier transfer between different task conditions on classification performance and physiological interpretability of results. Furthermore, we visualize the xDAWN spatial filter, which is especially well-suited for ERP components (see for example for P300 activity Rivet et al., 2009, 2012), to investigate the spatial distribution of relevant features used for single-trial detection. Finally, the obtained results are discussed in the scope of their relevance for neuroscience and BCI research as well as different application domains such as rehabilitation robotics.

## Method

### Ethics statement

The study has been conducted in accordance with the Declaration of Helsinki and approved with written consent by the ethics committee of the University of Bremen. Subjects gave informed and written consent to participate.

### Experimental design

**Figure 1:**
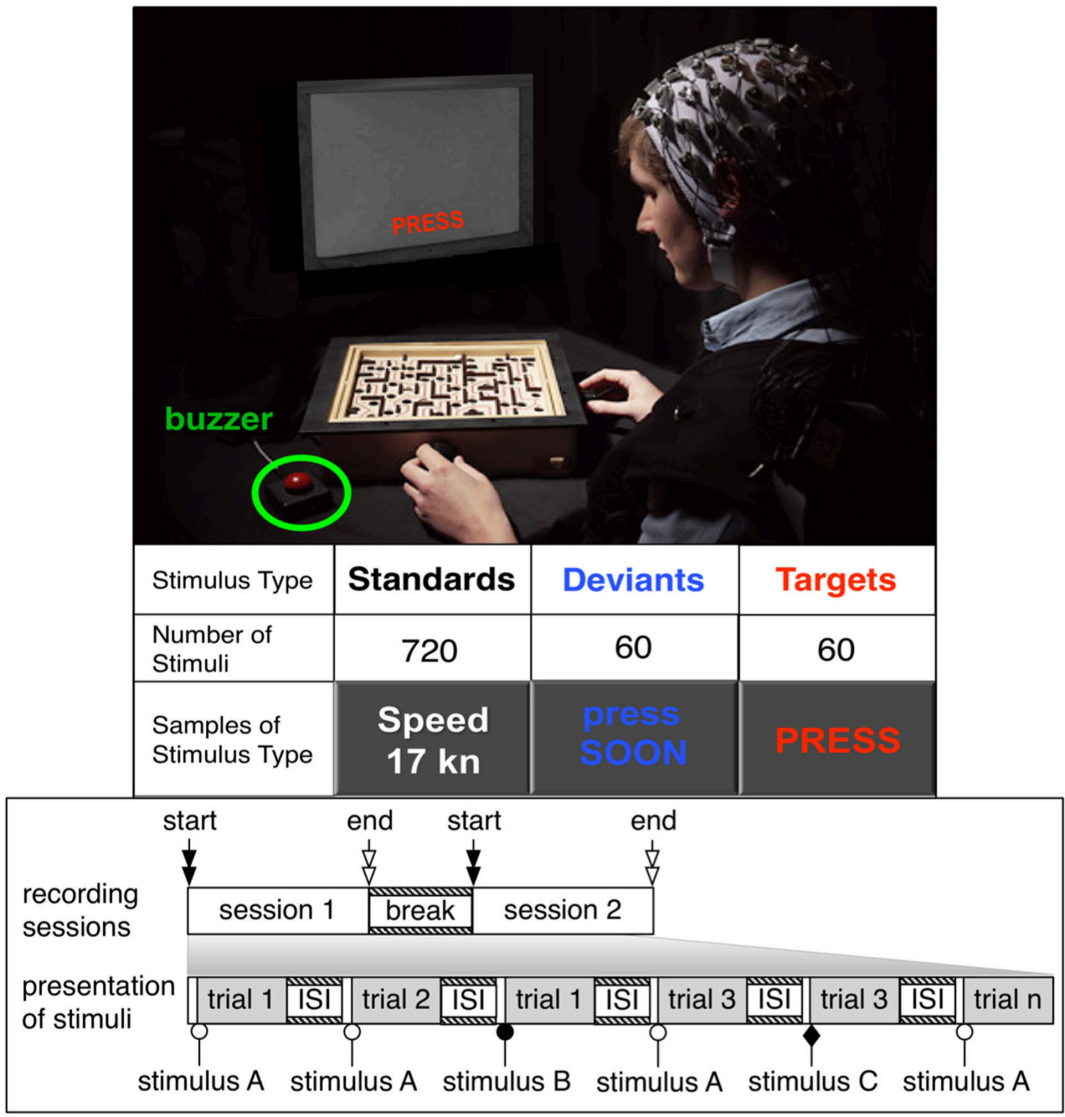
Experimental design “Labyrinth Oddball.” The upper part of the figure shows a subject performing in the experimental setup. Types and number of stimuli presented, session design is indicated in the lower part of the figure. The individual subject has given written informed consent to publish this image.

Since the intended investigation is not very straightforward in a complex eBR interaction application during the control of, e.g., several robots (Kirchner et al., 2016) or within a complex rehabilitation scenario (Seeland et al., 2017), the *experimental setup “Labyrinth Oddball”* was developed that allows to investigate ERP activity evoked by task-relevant and task-irrelevant stimuli (see Figure 1). Our experimental setup makes use of a *visual oddball discrimination paradigm* (Picton, 1992) which can either be performed without an interfering task (*simple task*: simple oddball task) or while performing a continuous sensor-motor, i.e., manipulation task (*dual task*: oddball task *while* playing a labyrinth game). In our case, the oddball task is the primary task instead of the secondary task in dual-task experiments for workload estimation (Isreal et al., 1980; Fowler, 1994; Kramer et al., 1995). The oddball task requires a response by the subject on infrequent task-relevant stimuli (targets) while ignoring *infrequent* task-irrelevant (deviants) and *frequent* task-irrelevant (standards) visual stimuli. In the developed experimental setup, infrequent *task-relevant* stimuli are expected to evoke a P3b and *infrequent* task-irrelevant stimuli are expected to evoke a P3a (Kok, 2001; Polich, 2007). We assume that the P300 component on infrequent *task-irrelevant* stimuli is smaller in amplitude compared to the P300 component evoked by infrequent *task-relevant* stimuli under both task conditions (simple task and dual task) caused by differences in task relevance or overlaying additional ERP activity. Additional overlaying ERP activity is supported by results of our former studies in complex BR applications mentioned above, where we found an unexpectedly strong expression of parietal positive ERP activity under multi-tasking conditions (e.g., Kirchner et al., 2013). In our study, under the dual-task condition, the subjects will also perform an additional ongoing task, i.e., to play the labyrinth game while remembering to respond when a specific stimulus, i.e., the task-relevant target stimulus, is presented to them and to finally execute the response behavior when the task-relevant target stimulus is recognized.

In the presented study thirteen subjects (two female, 11 male, age between 27 and 39 years; Master or PhD students, right-handed; normal or corrected-to-normal vision) participated in the experiments (see Figure 1). Each subject performed two experiments: a simple task and a dual task within two counterbalanced sessions that were performed the same day. In each session, subjects performed an oddball task and responded to infrequent task-relevant target stimuli (randomly mixed among frequent standard and infrequent deviant stimuli with a ratio of 1:12:1 and an Inter-Stimulus Interval (ISI) of 900 and 1, 100 ms) by pressing a buzzer. The type and number of stimuli presented per stimulus type and per task condition are depicted in Figure 1. Frequent task-irrelevant standard stimuli were words in white color (such as “speed 17 kn”) that gave irrelevant information, infrequent task-relevant target stimuli were the display of the word “PRESS” (displayed in red color), infrequent task-irrelevant deviant stimuli were the display of the word “press SOON” (displayed in orange color). Each stimulus was presented for 100 ms on a monitor that was placed directly behind the labyrinth game. Only in case that a task-relevant stimulus was presented, the subject had to press a buzzer which was located approximately 30 cm away from the left side corner of the game board.

During the *simple-task* condition, subjects were asked to hold both wheels of the labyrinth game that are designed to play the game (see below) while focusing on a ball placed in the middle of the game board; whereas during the *dual-task* condition, they were requested to play the labyrinth game (Figure 1) simultaneously (in addition to performing the oddball task). The labyrinth game consists of a maze with a track (marked by a continuous line on the game board) along which the player should maneuver a ball by tilting the board around two axes that are positioned perpendicularly to each other. Each axis can be turned by a wheel that is positioned on the right and left hand side of one corner of the game. Walls and holes are positioned along the trace. The walls can be used by the player to stay on the trace or to temporarily keep the ball in a safe position (e.g., in the corner between two walls). The holes must be avoided. Whenever the ball falls into a hole the player has to start again. The trace leads the player to a goal. Whenever the goal is reached the player has to move the ball back to the start position to play again. Playing the labyrinth game is an ongoing task in our experiments. It requires continuous and demanding senso-motor activity of the subject, namely to keep moving the ball through the maze while avoiding to lose the ball in one of the holes. To respond to a target stimulus during the dual-task condition the player has to let go of the left wheel to press a buzzer positioned on the left hand side of the labyrinth game. We chose the left hand to respond to buzzer press since it is more comfortable for the subject. Especially in case where the subject is putting the ball back to start position it would be impossible to respond with the right hand in case of a target event. For this reason only right handed subjects were chosen to attend the experiments. A run was finished based on the number of presented stimuli (a total of 60 targets, 60 deviants and 720 standards were presented under each condition) not on the quality of the play or progress in the labyrinth game.

All subjects that participated in the experiments were interviewed beforehand on how well they were able to play the game. All subjects performed a training session on a different day in which they only trained to play the game. Subjects that reported to have never played the game before were provided with a game for practical training several days to weeks before the experiments. Training progress was controlled by interviews. This procedure was chosen to assure that subjects would not be overstrained with the complicated senso-motor task with the unwanted result that they might give up to perform the game during the dual-task condition.

### Data recording

EEGs were recorded with 62 active electrodes (extended 10-20 actiCap system) and amplified by two 32-channel BrainAmp DC amplifiers (Brain Products GmbH, Munich, Germany). Electrodes were referenced to electrode FCz. Impedance was kept below 5 kΩ. Sampling rate was set to 2, 500 Hz and data was bandpass filtered between 0.1 Hz to 1, 000 Hz.

### Analysis of behavioral data

We analyzed subject's performance on correct behavior (response on target stimuli) and incorrect behavior (commission error: response on deviant stimuli and standard stimuli, and omission error: missing response on target stimuli). In case of correct behavior (response on target stimuli) response time was analyzed for both the simple-task and the dual-task condition as time between onset of stimulus presentation and the buzzer event.

### Analysis of event related potential (ERP)

EEGs were analyzed off-line with Brain Vision Analyzer Software Version 2.0 (Brain Products GmbH, Munich, Germany). First, EEGs were re-referenced to an average reference and filtered between 0.1 Hz and 30 Hz. Segments from 100 ms before to 1, 000 ms after stimulus onset were averaged based on stimulus of interest. Segments containing artifacts were rejected semi-automatically (amplitude > 100 and < −100 μV, gradient > 75μV). Target trials required a response within 200–2, 000 ms after stimulus onset to be counted as successful target trials. Only these successful target trials were used for calculating the average activity on target stimuli. By manual inspection it was found that the broad parietal ERP complex evoked by infrequent target stimuli and infrequent deviant stimuli showed differences between conditions (simple task vs. dual task) in a later time window starting at about 600 ms, while such differences were not so obvious for an earlier time window starting at 350 ms. Since the highest positive amplitudes were found in the earlier time window (before 600 ms) the late positive ERP complex was analyzed within two time windows (*early*: 350–600 ms; *late*: 600–850 ms) with respect to its maximum in amplitude. The definition of two time windows for maximum positive peak estimation was required to allow to analyze the observed differences in the later time window (absolute maximum peak amplitude was reached in the earlier time window). ERP activity before 300 ms was not investigated since we found in a similar experimental setting as used here that a classifier trained on ERP activity from early time windows, i.e., 0 ms to 350 ms after stimulus presentation was performing significantly worse (Kirchner et al., 2013). Thus, earlier activity was not assumed to be too relevant for single trial detection and therefore not investigated here. Besides, this work focuses on later positive parietal ERP components.

### Single-trial detection using machine learning

#### Dataset

One dataset for each task condition was recorded from 13 subjects. Each set contained 60 targets, 60 deviants, and 720 standards. However, for single-trial classification we used less than 60 examples for the target and deviant class and less than 720 examples for the standard class for each subject for two reasons:

1. Only artifact-free trials (as detected by artifact rejection for average ERP analysis) were used to make results of single-trial classification comparable with results of average ERP analysis
2. Only examples with correct behavior (i.e., with response on targets and no response on deviants) were used.

We merged datasets across all subjects for each task condition into one dataset and thus we obtained one dataset for simple-task and one set for dual-task condition. This procedure was chosen to minimize the effect of subject specificity and to compensate for different numbers of training examples for different subjects. Here, the total number of examples for each stimulus type was slightly different depending on each task condition (*simple task*: 7,000 trials for standard class, 463 for target class, 628 for deviant class; *dual task*: 6,255 for standard class, 363 for target class, 535 for deviant class). This procedure was chosen again to make results of single-trial classification comparable with results of average ERP analysis. Single trial detection analysis was performed using pySPACE (Krell et al., 2013).

#### Preprocessing

The continuous EEG signal was segmented into epochs from 0 to 1 s after each event type (standard/deviant/target). All epochs were normalized to zero mean for each channel, decimated to 25 Hz, and bandpass filtered between 0.1 and 4 Hz. The low-pass filter was used to assure that the classifier could only make use of data in the frequency range of mainly ERP activity. For a fair comparison with the results of average ERP analysis, the classifier used for single-trial detection was trained on EEG trials that were also used for average ERP analysis. Thus, only artifact-free trials were used. Moreover, we merged the data set across all subjects for each task condition to minimize subject-specific effects. This was especially relevant for the performed investigations of classifier transfer between task conditions.

#### Feature extraction

We used data points in the time domain as features. For multi-class classification (distinction between standards, deviants, and targets), we used all available 62 channels for feature extraction. For binary classification (standards vs. deviants, standards vs. targets, deviants vs. targets), besides using all available 62 electrodes, the definition of alternative electrode constellation was based on the characteristics of P300 (e.g., a maximum of averaged P3b was observed at electrodes Pz and CPz). Therefore, we chose a set of only these two parietal electrodes and a more extended set of parietal electrodes around Pz and CPz. In total, three electrode constellations were used to extract features: (a) All electrodes (62 channels), (b) Six parietal electrodes (CPz, CP3, CP4, Pz, P3, P4), and (c) Two parietal electrodes (CPz, Pz). For both binary and multi-class classification, the xDAWN (Rivet et al., 2009) was used as a spatial filter to enhance the signal-to-noise ratio, since it is especially suited for the analysis of EEG activity in the ERP domain (Rivet et al., 2009, 2012). After applying the xDAWN algorithm, the physical channels were reduced to the different numbers of pseudo-channels depending on electrode constellation (62 channels → 8 pseudo-channels, 6 channels → 1 pseudo-channel, 2 channels → 1 pseudo-channel). For the comparison between single-trial classification and ERP activity, we used two different time windows for feature extraction to investigate the different pattern of the late parietal positive ERP complex between the simple and dual task: early time window (400–600 ms) and late time window (600–800 ms). Features were extracted from 8 pseudo-channels (8 channels × 6 data points = 48) for each window and 1 pseudo-channel (1 channel × 6 data points = 6) for each window, respectively.

#### Classification

The features were normalized over all trials and used to train the classifier. We used a linear support vector machine (SVM) (Chang and Lin, 2011) for classification. For multi-class classification (distinction between standards, deviants, targets), one classifier was constructed per class pair (one-vs-one approach). In total, three classifiers were built and three classification results were aggregated to one final result. The class with the largest number of votes (the highest aggregated classification confidence, i.e., highest scores) was selected. For evaluation, we calculated the rates of correct classified instances of each class, i.e., true positive rates [TPR = TP/P, where P is the number of real positive instances (i.e., P = TP + TN)]. Note that each class has true positives (e.g., standard predicted as standard) and two types of false negatives (e.g., standard predicted as target (TN target) or deviant (TN deviant)). As a performance metric, we used the arithmetic mean of TPR of each class, i.e., (TPR standard + TPR target + TPR deviant)/3. We also calculated accuracy (ACC), i.e., (TP standard + TP target + TP deviant)/(TP standard + TP target + TP deviant + TN standard + TN target + TN deviant). However, ACC is sensitive when the ratio of classes is unbalanced (in case of imbalanced dataset). Hence, we used the arithmetic mean of TPR of each class (bACC), which is less sensitive to data with an unbalanced ratio of classes (details, see Straube and Krell, 2014). Additionally, we performed binary classifications for each class pair: target vs. standards, deviant vs. standards, target vs. deviants. Again, we used bACC, i.e., the arithmetic mean of the rate of correct classified instances of each class (positive or negative class), i.e., true positive rate (TPR) and true negative rate (TNR), where the *target* or *deviant* trials were the positive class.

For the no-transfer case (i.e., the case of no transfer between simple and dual task), a stratified 10 × 10 fold cross validation was used for evaluation. For each training, the cost parameter of the SVM (i.e., regularization constant Schölkopf et al., 2000) was optimized with a stratified five-fold cross validation using a grid search among the predetermined values (10^0^, 10^−1^, …, 10^−6^). Note that only 9 splits that were used for training were divided into 5 splits for the parameter optimization of the SVM. Due to the unbalanced ratio of *target* and *standard* trials (1:12) as well as *deviants* and *standard* trials (1:12), different penalty constants were used for the different classes (Veropoulos et al., 1999). We determined a class weight of 2 for the under-represented class as penalty. Hence, making errors on under-represented instances was costlier than making errors on over-represented instances. For the transfer case, the classifier trained on each task condition was used for evaluation. For example, the classifier trained on the data from simple task was used to evaluate the data from dual task. Hence, two different classifiers were build to detect single-trial ERP detection and there are two types of transfer cases: (a) simple task → dual task and (b) dual task → simple task.

### Statistical analysis

#### Behavior performances

Two different types of response behaviors were expected: (a) Commission errors (response on deviant stimuli) and (b) Omission errors (“missed targets,” i.e., absence of response after target stimuli). We observed only two commission errors for two subjects in total under the dual-task condition and none for the simple-task condition. Thus, we did not analyze the commission errors statistically. The omission errors were analyzed for each task (simple/dual) by Wilcoxen test. To evaluate the response time on target stimuli, the median of response time for each subject was estimated. Note that the median of response time had to be calculated, since the response times were not normally-distributed for all subjects. The median value of each subject was averaged across subjects for each task and between-task difference was tested by paired *t*-test.

#### Averaged ERP

To investigate the topography of the expected parietal positive broad ERP complex for each task condition, and furthermore to find out how the two different tasks (simple task, dual task) influence the expected positive broad parietal ERP complex, the positive peak amplitude of the mean ERP activity within two time windows (350–600 ms and 600–850 ms) was measured for each subject. The beginning and the end of each window were chosen based on: (a) the observed shape of averaged ERP activity on subject level and (b) the chosen procedure to divide the observed window of interest into two windows of the same size. Activity after 850 ms was not investigated to avoid overlap in ERP activity of consecutive trials possibly caused by the jitter in ISI of 200 ms. Note that the choice of window size did slightly differ from single-trial detection analysis, caused by the procedure in preprocessing and feature generation during single-trial detection analysis. However, the border between both windows was in both cases (ERP and single-trial detection analysis) chosen at 600 ms. The average amplitude values were analyzed across subjects by repeated measures ANOVA with four within-subjects factors: (a) Stimulus type (three levels: standards, targets, deviants), (b) Time window (two levels: 350–600 ms vs. 600–850 ms), (c) Parietal electrode: (three levels: CPz, Pz, POz), and (d) Task condition: (two levels: simple task, dual task). Electrodes CPz, Pz, POz were chosen, since the maximum amplitude of P3b was expected at electrode position Pz (Polich, 2007), while CPz was the next more frontal and POz the next more occipital central electrode to Pz. If necessary, Greenhouse-Geisser correction was applied. For pairwise comparisons Bonferroni correction was applied. Additionally, the normalized amplitude values based on Urbach and Kutas (2002) were analyzed with the same statistical design. Note that the statistical analysis on missed targets was not performed, since not all subjects missed targets in both task conditions.

#### Single-trial detection performances

Multi-class classification performances were analyzed by repeated measures ANOVA with two within-subjects factors: (a) time window (two levels: early window/late window), (b) task condition (two levels: simple task/dual task). Binary classification performances were analyzed by repeated measures ANOVA with four within-subjects factors: (a) time window (two levels: early window/late window), (b) task and transfer condition (four levels: simple task/dual task/dual task → simple task/simple task → dual task) (c) ERP detection type (three levels: target detection (t/st: targets vs. standards)/deviant detection (d/st: deviants vs. standards)/target detection (t/d: targets vs. deviants), and (d) number of channels used for training and testing (three levels: all 62 channels, 6 parietal channels/2 parietal channels). In the *post-hoc* tests, all levels of factors were compared (see Figures S3–S7). Again, Greenhouse-Geisser correction was applied where necessary. For pairwise comparisons, Bonferroni correction was applied.

## Results

### Behavioral performances

**Figure 2:**
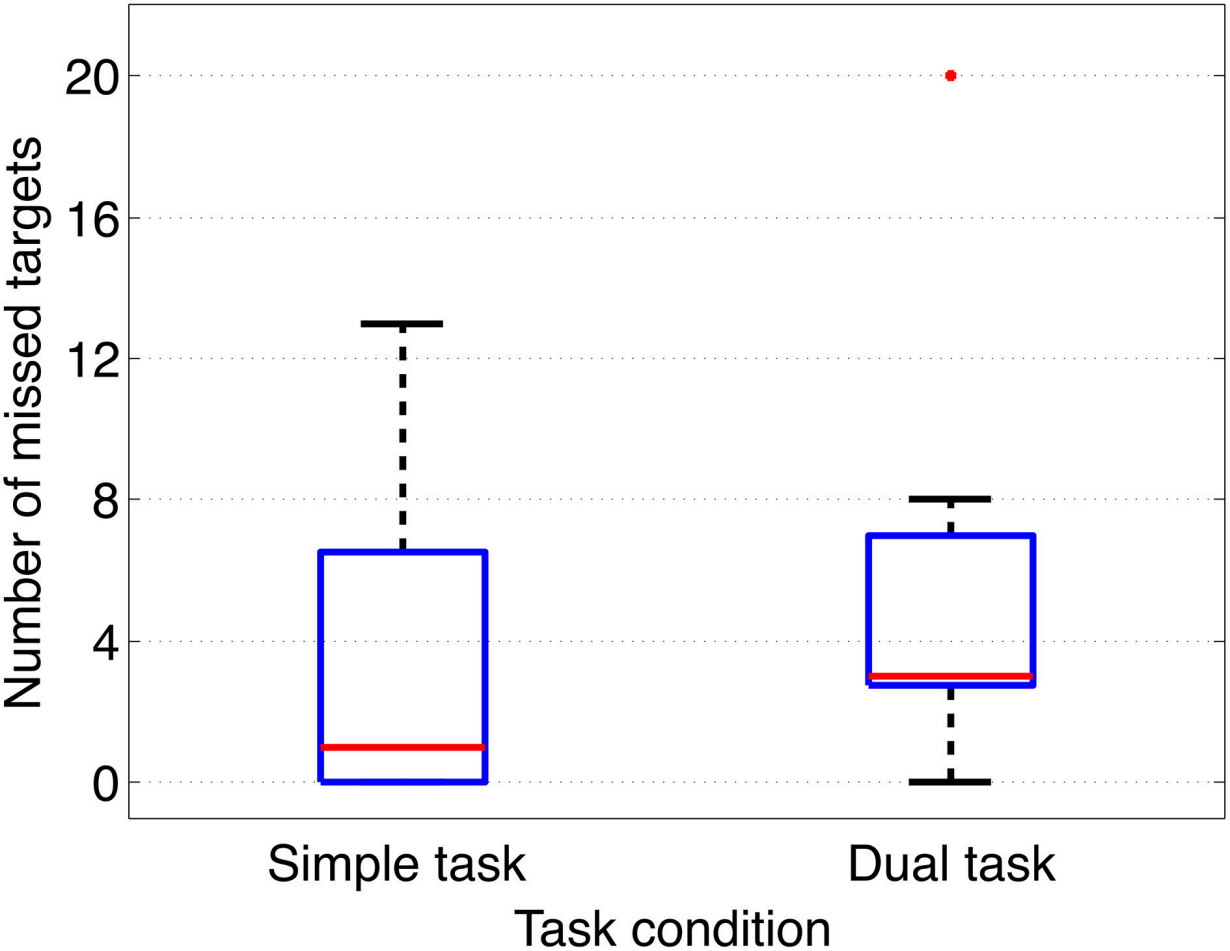
Omission errors. Omission errors (number of missed targets) under both task conditions (simple task and dual task) are illustrated.

**Figure 3:**
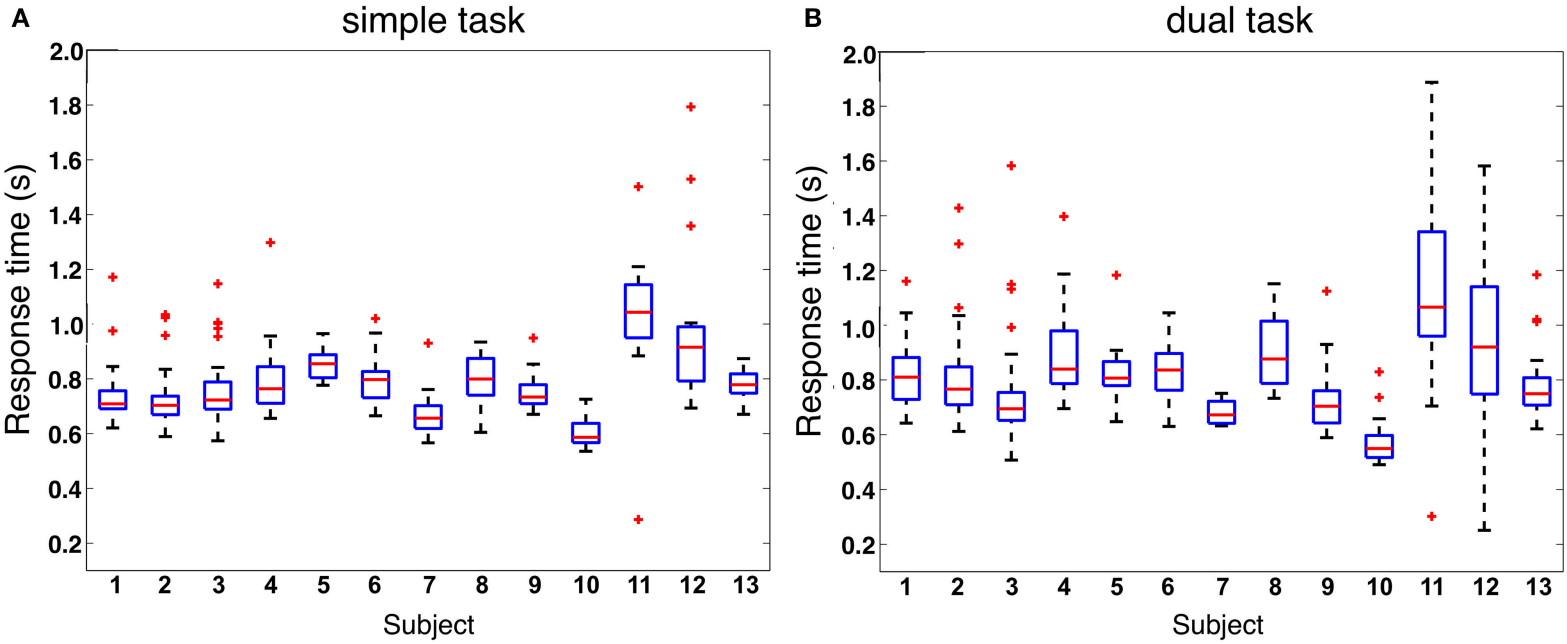
Response time. **(A)** response time on oddball target stimuli under simple-task condition (subjects are performing the oddball task only). **(B)** Response time on oddball target stimuli under dual-task condition (subjects are performing the oddball task while the ongoing task is to play the labyrinth game).

When analyzing response behavior we found only two commission errors for two subjects in total under the dual-task condition and none for the simple-task condition. Further, we found in total 50 (Mean: 3, 85, SD: 4, 69) omission errors under the simple-task condition and 67 (Mean: 5, 15, SD: 5, 06) omission errors under the dual-task condition (see also Figure 2). There was no significant difference between both task conditions. Figure 3 illustrates the median of response time on target stimuli for each subject under both task conditions (simple vs. dual-task condition). First, the median of response time was calculated for each subject and each task. The median values of each subject were normally distributed for each task. To compare between both tasks the median values were averaged across all subjects resulting in an averaged median value of 0.79 s for the simple oddball task (simple task) and of 0.77 s for the labyrinth oddball task (dual task). There was no significant difference in response time between both tasks [*t*_(12)_=−1.25, *p*=0.23].

### Averaged ERP

**Figure 4:**
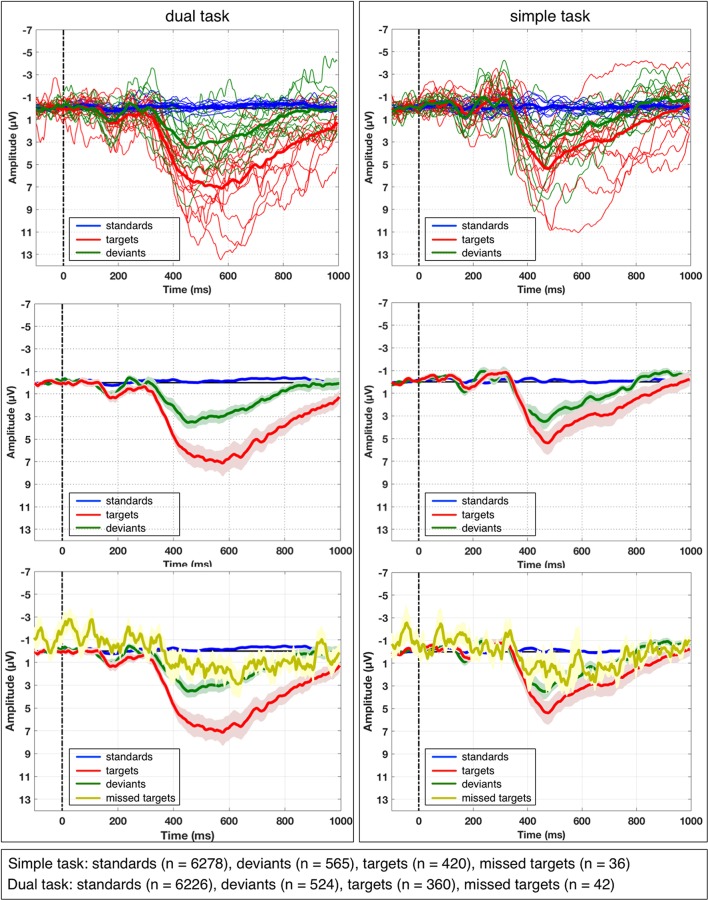
Average ERP and grand average of ERP activity at electrode Pz under both task conditions. Averaged ERP for each subject over all single-trials (thin lines) and grand averaged ERP over all subjects (thick lines) are depicted on different types of visual stimuli (standards, targets, and deviants) under simple-task condition and dual-task condition. A broad, sustained positive activity starting at 300 ms was observed at parietal sites. This activity was stronger for target stimuli compared to deviant stimuli. Additionally, standard error of mean (SEM) of grand average ERP is shown (in the middle), which was used for statistical analysis (pairwise comparisons). In the upper images, grand average of ERPs on missed targets are depicted for both task conditions.

**Figure 5:**
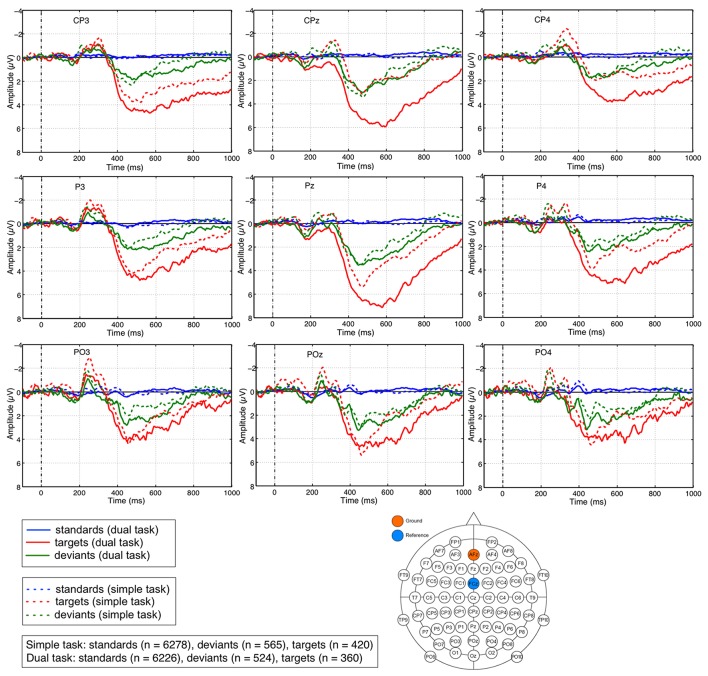
ERP activity at individual parietal electrodes. Grand averages of ERP activity evoked at electrodes Pz, CPz, POz, P3, P4, CP3, and CP4 show a broad sustained ERP activity on deviant and target stimuli under both task conditions (simple and dual task) starting at 350 ms. Most prominent differences in the amplitude of the parietal positivity on targets compared to deviants can especially be observed for the late time window (starting at 600 ms) under dual-task condition.

Figure 4 illustrates the average ERPs for each subject and the grand average ERPs across all subjects on the three different types of visual stimuli (standards, targets, deviants at electrode Pz for each task (*simple* and *dual-task*). Additionally, grand average ERPs across all subjects on missed targets are depicted in Figure 4 lower part. We observed a similar pattern in the grand average ERP on missed targets in both task conditions. We obtained the grand averages by averaging the average curves of all participating subjects for each task condition separately. Figure 5 depicts grand average ERPs at parietal electrodes CPz, Pz, POz, P3, P4, CP3, and CP4. Average ERPs of individual subjects are illustrated in Supplementary Materials in Figure S1.

We observed a maximum positive broad ERP complex at parietal sites which was evoked by infrequent task-relevant *target* stimuli as well as by infrequent *deviant* stimuli if compared to frequent *standard* stimuli. These positive ERP activities evoked by targets and deviants were found under both task conditions (*simple task* and *dual task*), for all three investigated electrodes (*CPz, Pz, POz*) and for both time windows (*early window* and *late window*).

The statistical evaluation shows a significant difference between three stimulus types [main effect: *F*_(2, 24)_=58.412, *p* < 0.001, standards vs. targets: *p* < 0.001, standards vs. deviants: *p* < 0.002, targets vs. deviants: *p* < 0.002]. The results of *post-hoc* analysis on differences between three stimulus types for each window, each electrode, and each task condition are reported next. Under *dual-task* condition for both windows at all three electrodes, there was a significantly higher amplitude of the broad positive ERP complex on deviants or targets compared to standards (early window: *p* < 0.001 for standards vs. deviants as well as standards vs. targets at all three electrodes; late window: *p* < 0.001 for standards vs. deviants at all three electrodes, *p* < 0.001 for standards vs. targets at CPz and Pz; *p* < 0.002 at POz, see Figure S2). Further, we observed significant differences between the amplitudes evoked by deviants and targets, i.e., a higher amplitude on targets compared to deviants (early window: *p* < 0.001 at Pz, *p* < 0.002 at CPz, and *p* < 0.005 at POz; late window: *p* < 0.001 at Pz, *p* < 0.002 at CPz, and *p* < 0.023 at POz, see Figure S2). Under *simple-task* condition, we found again a significantly higher amplitude of the broad positive ERP complex on deviants or targets compared to standards in the early window (standards vs. targets: *p* < 0.005 at all electrodes; standards vs. deviants: *p* < 0.001 at all electrodes, see Figure S2). For the late window, a significantly higher amplitude of the broad positive ERP complex on deviants or targets compared to standards were found as well except for CPz in the comparison between standard and target (standards vs. targets: *p*=*n*.*s*. at CPz, *p* < 0.017 at Pz, *p* < 0.015 at POz; standards vs. deviants: *p* < 0.029 at CPz, *p* < 0.006 at Pz, *p* < 0.013 at POz). Moreover, significant differences between the amplitude evoked by deviants and targets, i.e., higher amplitude on targets compared to deviants were only found in the early window at electrodes Pz (*p* < 0.037) and POz (*p* < 0.002), but not for CPz (*p*=*n*.*s*) and not for any of the three parietal electrodes in the later window (*p*=*n*.*s*. for CPz, Pz, and POz).

Furthermore, we found a significant difference between both task conditions (main effect: *F*_(1, 12)_=6.846, *p* < 0.024). The results of *post-hoc* analysis on differences between both task conditions for each window, each electrode, and each stimulus type are reported as follows. We observed a significant difference between the simple and dual task only in the late window (simple task vs. dual task: *p* < 0.001 at CPz and Pz, *p* < 0.008 at POz, see Figure S2). This task-specific difference was observed in the positivity evoked by targets, but not by deviants (simple task vs. dual task: *p*=*n*.*s*., see Figure S2). In addition, we also observed the task-specific difference which was shown only for targets in the early window, but only at CPz and Pz (simple task vs. dual task: *p* < 0.001 at CPz, *p* < 0.003 at Pz, *p*=*n*.*s*. at POz, see Figure S2).

In summary, we found a positive ERP activity that was strongly evoked by targets than deviants for both time windows under dual-task condition. Such significant differences could not be found under simple-task condition for the late window and only at electrodes Pz and POz for the early window. Furthermore, we found a difference between both task conditions in the late window when the positive ERP activity was evoked by targets, but not by deviants. A similar pattern was also shown in the early window except for CPz.

### Single-trial detection performances

Table 1 shows single-trial classification performances for the distinction between standards, deviants, and targets (multi-class classification). We achieved a high bACC of 0.73 under simple task for both early and late window (chance level: 0.33 bACC). Under the dual-task condition, we also achieved a higher performance for early window compared to late window [early window (0.71 bACC) vs. late window (0.67 bACC): *p* < 0.001]. The classification performance was higher for the simple-task condition compared to the dual-task condition for both windows (simple task vs. dual task: *p* < 0.001 for both windows).

**Table 1:** Multi-class classification performance.

	**Simple task**			**Dual task**		
	**Standard**	**Target**	**Deviant**	**Standard**	**Target**	**Deviant**
**EARLY TIME WINDOW (0.4–0.6 s)**						
TPR	0.93 ± 0.03	0.77 ± 0.07	0.50 ± 0.09	0.88 ± 0.04	0.71 ± 0.08	0.54 ± 0.08
bACC	0.73 ± 0.03			0.71 ± 0.03		
ACC	0.89 ± 0.02			0.84 ± 0.04		
**LATE TIME WINDOW (0.6–0.8 s)**						
TPR	0.90 ± 0.03	0.80 ± 0.06	0.48 ± 0.07	0.91 ± 0.04	0.69 ± 0.10	0.42 ± 0.10
bACC	0.73 ± 0.03			0.67 ± 0.04		
ACC	0.86 ± 0.02			0.86 ± 0.03		

*Mean and standard error of mean were reported. TPR = TP/TP+FN (e.g., TP: standard predicted as standard; TN: standard predicted as target or deviant), bACC = (TPR standard + TPR target + TPR deviant)/3. ACC = (TP standard + TP target + TP deviant)/(TP standard + TP target + TP deviant + TN standard + TN target + TN deviant). Details for metrics, see text. Note that 62 channels were used for classification (Details, see text)*.

**Figure 6:**
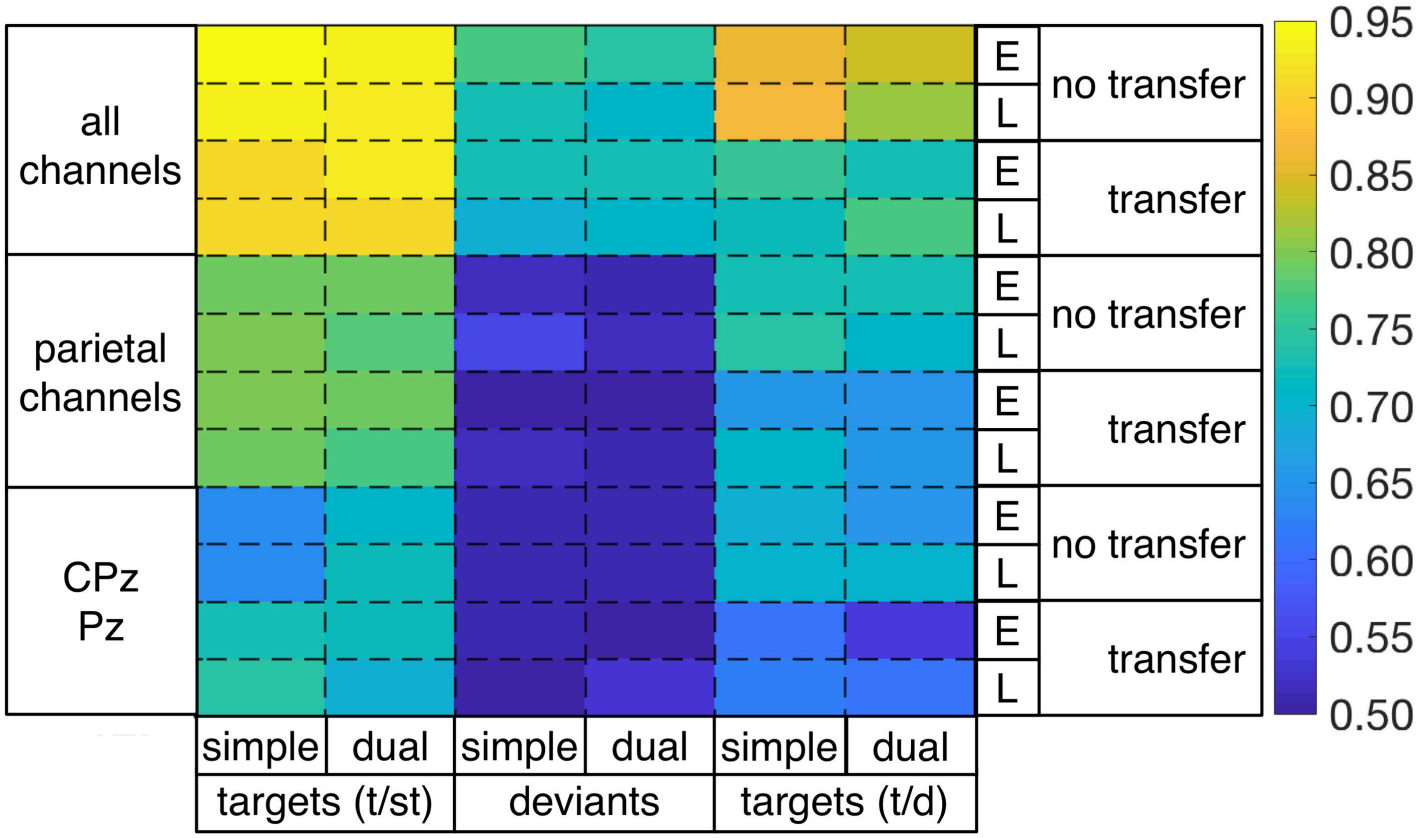
Performances in single-trial detection of targets [targets vs. standards (t/st) or targets vs. deviants (t/d)] and deviants [deviants vs. standards (d/st)] for both task conditions (simple/dual), both windows (early/late), and both no-transfer case and transfer case in case of using all 62 channels, 6 parietal channels (CPz, CP3, CP4, Pz, P3, P4), or 2 parietal channels (CPz, Pz) for classification (E: early window, L: late window).

Table 2 shows single-trial classification performance (bACC) on two types of target detection and deviant detection: target detection (t/st: targets vs. standards), deviant detection (d/st: deviants vs. standards), target detection (t/d) targets vs. deviants. Figure 6 illustrates classification performance as given in Table 2 over all conditions. Blue areas indicate balanced accuracy (bACC) at chance level (0.5 bACC). We obtained a high single-trial detection of both target detection and deviant detection under both task conditions and both windows when using all 62 channels [target detection (t/st): 0.95 to 0.93, deviant detection (d/st): 0.77 to 0.71, target detection (t/d): 0.87 to 0.81; chance level: 0.5)]. We found a higher performance on single-trial detection of target detection compared to deviant detection for both windows and both task conditions in case of using 62 channels [target detection (t/st) vs. deviant detection: *p* < 0.001, target detection (t/d) vs. deviant detection: *p* < 0.001, see color changes between target detection and deviant detection, e.g., target detection (t/st): light-yellow, target detection (t/d): orange, deviant detection: green, also see Figure S3, where statistical values for comparison between target (t/st or t/d) and deviant detection are reported]. Between both types of target detection, the performance of target detection was higher when the classifier trained on targets and standards was used for target detection [target detection (t/st) vs. target detection (t/d): *p* < 0.001, statistical values for comparison between both types of target detection, see Figure S3].

**Table 2:** Binary classification performance (no transfer between task conditions). Mean and standard error of mean are reported.

	**Early time window (0.4–0.6 s)**		**Late time window (0.6-0.8 s)**	
	**Simple task**	**Dual task**	**Simple task**	**Dual task**
(A) Single-trial detection of **targets** (distinction of **targets vs. standards**)				
All channels	0.95 ± 0.01	0.94 ± 0.01	0.94 ± 0.01	0.93 ± 0.01
Parietal channels	0.79 ± 0.01	0.79 ± 0.01	0.80 ± 0.01	0.78 ± 0.01
CPz,Pz	0.64 ± 0.01	0.71 ± 0.01	0.64 ± 0.01	0.72 ± 0.01
(B) Single-trial detection of **deviants** (distinction of **deviants vs. standards**)				
All channels	0.77 ± 0.01	0.75 ± 0.01	0.73 ± 0.01	0.71 ± 0.01
Parietal channels	0.52 ± 0.01	0.51 ± 0.01	0.55 ± 0.01	0.52 ± 0.01
CPz,Pz	0.51 ± 0.01	0.51 ± 0.01	0.51 ± 0.01	0.51 ± 0.01
(C) Single-trial detection of **targets** (distinction of **targets vs. deviants**)				
All channels	0.86 ± 0.01	0.84 ± 0.03	0.87 ± 0.03	0.81 ± 0.04
Parietal channels	0.73 ± 0.04	0.73 ± 0.04	0.74 ± 0.04	0.71 ± 0.04
CPz,Pz	0.69 ± 0.04	0.65 ± 0.05	0.70 ± 0.01	0.67 ± 0.01

**Figure 7:**
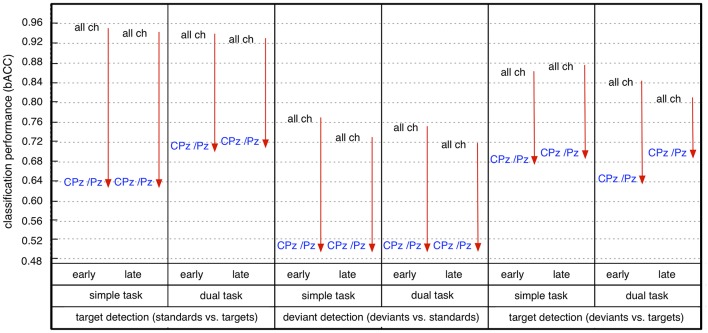
Effect of reduction of channel number on classification performance (early: early window, late: late window, all ch: all 62 channels, CPz/Pz: 2 parietal channels). Arrow direction refers to significant increases or decreases. Lines without arrow direction refer to no significant difference. Statistical values for comparison are reported in Supplementary Materials in Figure S4 (comparison between *all* 62 *channels (all Ch)* and 2 *parietal channels (CPz/Pz)* in Figure S4).

Furthermore, we found that the reduction of the number of channels had an impact on classification performance (see Figure 7). The significant reduction of target detection (t/st and t/d) due to reduced number of channels was shown for both windows and both task conditions [statistical values for comparison among 62, 6, and 2 channels for both types of target detection (t/st and t/d), see Figure S4]. However, target detection was still high in case of using 6 electrodes [target detection (t/st): 0.80–0.78, target detection (t/d): 0.74–0.71, see light-green areas for target detection (t/st and t/d) in Figure 6] and well above chance level in case of using 2 parietal electrodes [see green or light-blue areas for target detection (t/st or t/d) in Figure 6, see also Figure 7]. Such reduction was also shown in deviant detection, but there was no significant difference between 6 and 2 channels, since a reduction to both 6 and 2 parietal electrodes led to classification performance at chance level [statistical values for comparison among 62, 6, and 2 channels for deviant detection (d/st), see Figure S4].

**Figure 8:**
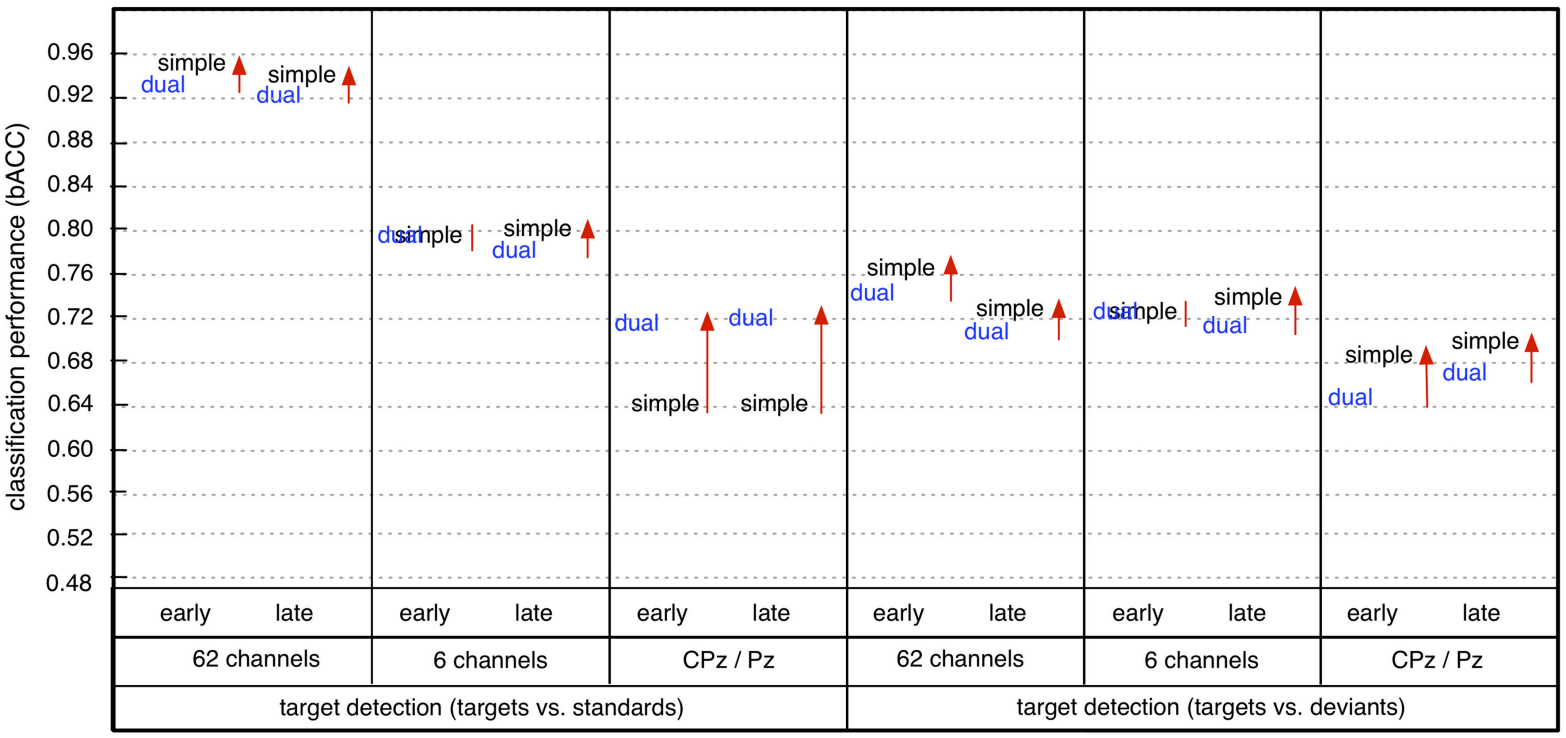
Effect of task condition on classification performance (early: early window, late: late window, simple: simple task, dual: dual task). Arrow direction refers to significant increases or decreases. Lines without arrow direction refer to no significant difference. Statistical values for comparison are reported in Supplementary Materials in Figure S5 (comparison between *simple task (s)* and *dual task (d)* in Figure S5).

This effect of number of channels on classification performance was different depending on task condition (i.e., task-specific effect). In case of using all 62 parietal channels, we found a significantly higher performance under the simple-task condition compared to the dual-task condition for both types of target detection (t/st and t/d) and deviant detection in the no-transfer case (see Figure 8, statistical values for comparison between task conditions, see Figure S5). In case of using 6 parietal channels, we found no performance difference between simple-task and dual-task condition for both target detection (t/st and t/d) and deviant detection in the no-transfer case (simple task vs. dual task: *p*=*n*.*s*., statistical values for comparison between task conditions, see Figure S5). In case of using 2 parietal channels, we found a significantly higher performance under the dual-task condition compared to the simple-task condition for target detection (t/st) in case of using a classifier trained on targets and standards, but not for deviant detection in the no-transfer case [simple task vs. dual task: *p* < 0.002 for target detection (t/st), *p*=*n*.*s*. for deviant detection, statistical values for comparison between task conditions, see Figure S5]. This superior target detection (t/st) performance under the dual-task condition thus showed the opposite pattern compared to using all 62 channels in the no-transfer case. For target detection (t/d) in case of using a classifier trained on targets and deviants, the same pattern as in the case of using 62 channels was observed, i.e., target detection was better under the simple-task condition compared to the dual-task condition in the no-transfer case (simple task vs. dual task: *p* < 0.001, statistical values for comparison between task conditions, see Figure S5).

**Figure 9:**
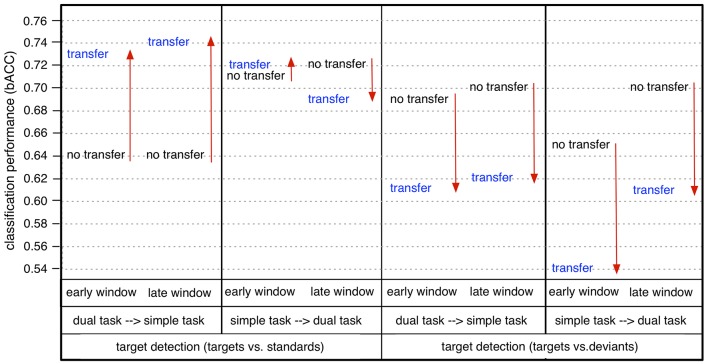
Comparison between no transfer and transfer in binary classification performance (bACC) in case of using 2 channels (CPz/Pz): dual task → simple task (transfer) vs. simple task (no transfer), simple task → dual task (transfer) vs. dual task (no transfer). Arrow direction refers to significant increases or decreases. Lines without arrow direction refer to no significant difference. Statistical values for comparison are reported in Supplementary Materials in Figure S5 (comparison between *dual task* → *simple task (d*→*s)* and *simple task (s)* and comparison of between *dual task* → *simple task (d*→*s)* and *dual task (d) in* Figure S5).

In case of task transfer we obtained the following findings (see Table 3). In case of using a classifier trained on targets and standards, target detection (t/st) was slightly reduced while using 62 or 6 channels (simple task: 0.95 → 0.91, dual task: 0.94 → 0.93 in case of using 62 channels, see also color changes from light yellow to dark-yellow area in Figure 6; simple task: 0.79 → 0.79, dual task: 0.79 → 0.77 in case of using 6 channels, see also Figure 6). However, when using only 2 electrodes, target detection (t/st) was strongly improved in the transfer direction from the *dual* task to *simple* task for both windows (early window: 0.64 vs. 0.73, *p* < 0.001, late window: 0.64 vs. 0.73, *p* < 0.001, see Figure 9, and also changes in color range blue to green in Figure 6, statistical values for comparison between transfer conditions, see Figure S5). In the reversed transfer direction, however, we did not observe such performance improvements in target detection (t/st) (early window: 0.71 → 0.72, *p*=*n*.*s*. late window: 0.72 → 0.69, *p*=*n*.*s*., statistical values for comparison between transfer conditions, see Figure S5).

**Table 3:** Binary classification performance (classifier transfer between simple and dual task).

(A) Transfer between simple and dual task: single-trial detection of **targets**		
(Classifier trained on *targets* and *standards* were used to detect *targets*)		
0.4–0.6 s	dual task (training) → simple task (test)	simple task (training) → dual task (test)
All channels	0.91 ± 0.01	0.93 ± 0.01
Parietal channels	0.80 ± 0.01	0.79 ± 0.01
CPz,Pz	0.73 ± 0.01	0.72 ± 0.08
0.6–0.8 s	Dual task (training) → Simple task (test)	Simple task (training) → dual task (test)
All channels	0.91 ± 0.01	0.91 ± 0.01
Parietal channels	0.79 ± 0.01	0.77 ± 0.01
CPz,Pz	0.74 ± 0.01	0.69 ± 0.10
(B) Transfer between simple and dual task: single-trial detection of **deviants**		
(Classifier trained on *deviants* and *standards* were used to detect *deviants*)		
0.04-0.6 s	dual task (training) → simple task (test)	simple task (training) → dual task (test)
All channels	0.73 ± 0.01	0.73 ± 0.01
Parietal channels	0.50 ± 0.01	0.50 ± 0.01
CPz,Pz	0.51 ± 0.01	0.50 ± 0.01
0.6–0.8 s	dual task (training) → simple task (test)	simple task (training) → dual task (test)
All channels	0.69 ± 0.01	0.71 ± 0.01
Parietal channels	0.52 ± 0.03	0.51 ± 0.04
CPz,Pz	0.50 ± 0.02	0.53 ± 0.01
(C) Transfer between simple and dual task: single-trial detection of **targets**		
(Classifier trained on *targets* and *deviants* were used to detect *targets*)		
0.4–0.6 s	dual task (training) → simple task (test)	simple task (training) → dual task (test)
All channels	0.76 ± 0.01	0.73 ± 0.01
Parietal channels	0.66 ± 0.01	0.65 ± 0.03
CPz,Pz	0.61 ± 0.01	0.54 ± 0.01
0.6–0.8 s	dual task (training) → simple task (test)	simple task (training) → dual task (test)
All channels	0.72 ± 0.01	0.77 ± 0.01
Parietal channels	0.71 ± 0.01	0.66 ± 0.01
CPz,Pz	0.62 ± 0.02	0.61 ± 0.01

*Mean and standard error of mean were reported*.

However, when using a classifier trained on targets and deviants, performance in target detection (t/d) was significantly reduced in case of both directions of transfer (see Figure 9, see also color changes (green to blue) in Figure 6). For deviant detection, there was no large effect of task transfer, i.e., we observed a slight difference between no-transfer case and transfer case for all types of channel constellation and both windows (see Figure 9, statistical values for comparison between transfer conditions, see Supplementary Materials in Figure S5).

In summary, we achieved a very high performance in both types of target detections and deviant detection in case of using 62 electrodes. Both types of target detection (t/st and t/d) were better than deviant detection for all conditions, i.e., for both windows, both task conditions, and all types of channel constellation. Further, target detection in case of using a classifier trained on targets and standards was superior compared to target detection when using a classifier trained on targets and deviants for all conditions. When using less electrodes (6 or 2 parietal electrodes), classification performance was reduced in general (see Figure 7). However, classification performance on target detection was still high compared to deviant detection which was at chance level in case of using 6 or 2 parietal channels. Especially, target detection (t/st) was extremely decreased due to reduced channel numbers under the simple-task condition, but not under the dual-task condition (see Figure 8). In fact, target detection (t/st) was better under the simple-task condition compared to the dual-task condition in case of using 62 channels. In contrast, in case of using 2 parietal channels (CPz/Pz), the opposite pattern was shown, i.e., target detection (t/st) was better under the dual-task condition compared to the simple-task condition. In particular, in case of using 2 parietal channels, a significant increase in target detection (t/st) in the transfer direction from *dual* task to *simple* task compared to the reversed transfer direction was found (see Figure 9).

### Spatial distribution by xDAWN filter

**Figure 10:**
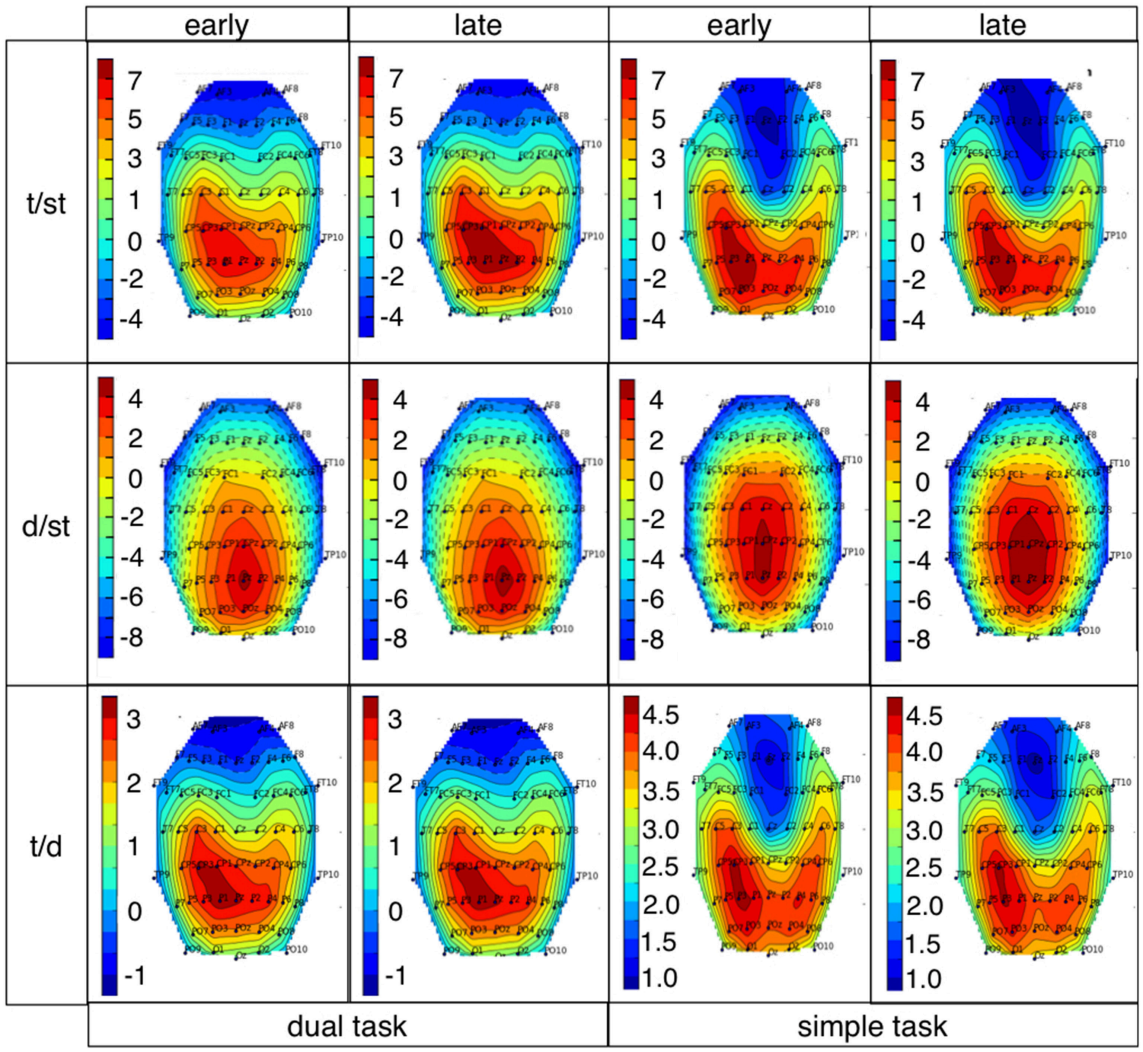
xDAWN images for target (t/st and t/d) and deviant detection for both task conditions and both windows (E: early window, L: late window, (t/st: target detection using a classifier trained on targets and standards, d/st: deviant detection using a classifier trained on deviants and standards, t/d: target detection using a classifier trained on targets and deviants). Details, see text.

Figure 10 shows the spatial distribution of the first retained channel from xDAWN spatial filter, which was used for training in case when all 62 recorded channels were retained. In general, we observed stronger weights for parietal area in case of target detection (t/st) compared to deviant detection. Further, slightly stronger weights were observed for the late window compared to the early window in case of target detection (t/st) under dual-task condition. In general, the spatial distribution was more spread over parietal and central area in case of deviant detection, while the spatial distribution in case of target detection was more focused on parietal area. This pattern was shown for both types of target detection (t/st and t/d). Target detection in case of training on targets and deviants (t/d) resulted in reduced weights of the first retained channel compared to target detection in case of training on targets and standards (t/st).

## Discussion

### Behavioral data

Results of behavioral data analysis indicate that deviant and target stimuli were successfully evaluated by the subjects under both task conditions with respect to their task-relevance, shown by very accurate performance in response behavior. Under both task conditions a very low number of commission errors was observed. The number of omission errors was not significantly different between both task conditions and relatively low (all subjects but one outlier under dual-task condition, see Figure 2). This finding indicates that workload was not strongly increased by adding the second task. This result is supported by the finding that response time was not significantly different between both task conditions. This is an important finding, since response latency was found to have an effect on the amplitude of the parietal ERP complex as shown in an earlier study (Kim and Kirchner, 2012 and Figures S7, S8 in Supplementary Materials). Hence, we can exclude effects of response time on the ERP expression.

### Average ERP analysis and results of spatial filter

Our results show that a positive parietal ERP complex was evoked by target as well as deviant stimuli under both simple-task and dual-task condition. Our findings are supported by the investigation of xDAWN filters that are relevant for the classifier to learn the differences between classes. Features for single-trial classification were chosen by xDAWN filter mainly from parietal electrodes.

While performing complex sensor-motor behavior during human-machine interaction (dual-task condition) a broader parietal positivity was elicited on infrequent task-relevant (target) events compared to infrequent task-irrelevant (deviant) events with higher amplitude at parietal electrodes in both investigated time windows. The same pattern was found under simple-task condition. Again, this finding can be supported by results of spatial filtering. xDAWN features for deviant trials are weaker than for target trials.

From literature we expect that P300 will contribute mainly to the investigated early time window (Kok, 2001; Polich, 2007). The stronger expression of the parietal positivity under simple-task condition in the *early time window* on *target* stimuli compared to non-target *deviant* stimuli is caused by differences in P300 expression, i.e., only targets will evoke a strong P3b due to the task-relevance. In our results, in the early time window P300 was not reduced under dual-task compared to simple-task condition. P300 is usually found to be reduced under dual-task compared to simple-task condition (Isreal et al., 1980; Kramer et al., 1983). However, in more complex application scenarios (e.g., multi-robot control Kirchner et al., 2016) that require dual or multi-tasking, we often found a strong and broadly expressed parietal ERP complex evoked by task-relevant stimuli. As introduced earlier, most studies that make use of the oddball paradigm typically make use of either (1) task-irrelevant *frequent* (standards) and task-irrelevant *infrequent* stimuli (deviants) Kramer et al. (1995) or (2) task-*irrelevant* frequent (standards) and task-*relevant* infrequent stimuli (targets) (Isreal et al., 1980; Kramer et al., 1983). There are only few studies combining (1) and (2) as for example in (Kramer et al., 1995) where subjects performed an oddball task responding only to one of two infrequent auditory stimuli interleaved with distinct frequent auditory stimuli as a control condition for the irrelevant-probe experiment. Here, the authors found significantly larger P300 amplitudes on task-*relevant* infrequent stimuli (targets) compared to task-*irrelevant* infrequent stimuli (deviants) at electrode Pz. Hence, task-relevance of infrequent stimuli (targets) might be more important for the expression of parietal ERP components than subjective rarity of stimuli. Task-relevance of the stimuli is known to influence the amplitude of the P3b (Kok, 2001; Polich, 2007). Under this combined condition (oddball making use of (1) deviants and (2) targets), we assume that not only targets but also task-irrelevant infrequent stimuli (deviants) will evoke a P3b (besides an overall weaker P3a on deviant stimuli), since deviants must be evaluated regarding their task relevance in case that both types of stimuli are difficult to distinguish. Hence, besides a P3a, a P3b might be evoked by task-*irrelevant* infrequent stimuli in case that task relevance is difficult to be evaluated. Results of spatial filtering support the finding that task-*irrelevant* infrequent stimuli (deviants) might to some degree require evaluation with respect to its task-relevance especially under dual-task condition requiring multi-motor tasking, since for deviant stimuli a more posterior feature contribution can be found under dual-task compared to simple-task condition. However, even under simple-task condition, spatial features were chosen by xDAWN from parietal sites with extension to central sites. This might result from the experimental instruction that subjects should not focus the text presented on the screen but focus the marble of the game at all time and hence had more difficulties to distinguish deviant and target stimuli.

Alternatively, the missing reduction of amplitude of the positive parietal ERP complex under simple-task compared to dual-task condition (for both deviant and target stimuli) could be explained by the increase of task emphasis of the subjects under the dual-task compared to the simple-task condition (see Kok, 2001; Miller et al., 2011 for discussion of factors influencing the amplitude of P3b). In our study, subjects consistently reported that the primary visual-motor oddball task in the simple task was boring and they had a hard time staying focussed on the task. Further, subjects reported that the dual task was more interesting and seemed to last shorter than the simple task. Other known influencing factors were eliminated by experimental design. By counterbalancing both conditions across subjects and by overtraining the subjects in playing the labyrinth game, a training effect on the amplitude of P3b could be excluded. Furthermore, we requested the subjects to focus on the ball under both simple-task as well as dual-task condition. Thus, stimuli from the visual oddball were presented in the periphery of the field of view under both task conditions. A possible influence of stronger visual focus on the oddball stimuli was excluded by this procedure Differences between task conditions are therefore also unlikely to result from eye artifacts, which were mostly avoided by the instruction given to the subjects. Additionally, EEG trials with eye artifacts (mainly from blinking) were excluded from ERP as well as xDAWN analysis. Further, a strong contribution of eye artifacts would have resulted in xDAWN features with strong contribution from frontal electrode sites. Such differences in xDAWN features could not be found.

While the found missing reduction of evoked parietal ERP activity on *deviant* stimuli can be explained as argued above, the increase of the amplitude of the evoked parietal ERP activity on *target* events deserves further discussion. For both time windows it was found that the ERP activity was enhanced on targets when the secondary task (playing the labyrinth game) was added (except for POz, see Supplementary Materials in Figure S2B). This was not found for deviant events (see Supplementary Materials in Figure S2B). Additionally, differences in the parietal broad positive ERP complex on *target* vs. on *deviant* stimuli in the *late time window* were only observed under the dual-task condition, not under the simple-task condition (see Supplementary Materials in Figure S2A). In our study, under dual-task condition subjects had to change their motor behavior from controlling the game to responding to the infrequent motor task-relevant (target) event only in case that a target event occurred but not in case that a deviant stimulus was presented. Our results indicate that complex senso-motor dual-tasking behavior not only requires target recognition processes that evoke a P3b (Kok, 2001; Polich, 2007), but additional processes such as brain processes that are correlated with task changes. For example, the retrieval of prospective memory (PM) and configuration of PM tasks will evoke a parietal positivity, the so-called prospective positivity in addition to a P3b (Bisiacchi et al., 2009; West, 2011). PM paradigms investigate processes that are associated with remembering to perform an intended action as soon as a cue (PM cue) is recognized that reminds the subject to perform the remembered task (see for example Bisiacchi et al., 2009). By combining a complex senso-motor task with a visual-motor discrimination task (both requiring a different type of motor response behavior) our study was designed to copy such a PM task situation to some degree: subjects were instructed to remember to respond to targets by pressing the buzzer, while he or she was playing the game. Hence, under dual-task condition task-relevant target stimuli might also function as PM cues and will evoke PM-related brain activity. In fact, a previous study (West et al., 2003) found that task-relevant stimuli evoke *both* target recognition *and* PM-related activity. In this study, it was found that the P3b contributes to the parietal positive ERP complex evoked in PM experiments. Their findings support that PM performance requires target recognition. It was also shown that the P3b is evoked earlier than the prospective positivity, which can be found in the time window between 600 and 800 ms and represents a later component of the parietal positive ERP complex that is evoked during PM performance. Furthermore, the prospective positivity was discussed as ERP that is clearly distinguishable from brain processes that are involved in the detection of infrequent target stimuli correlated with the P3b (West et al., 2003). Hence, our assumption is that other ERPs than P3b are evoked in complex multi-motor task applications, i.e., under dual-task condition of the labyrinth game. A strongly expressed and broad parietal positive ERP complex was found in all of our P300-based eBR applications (Kirchner et al., 2013; Woehrle and Kirchner, 2014a; Kirchner et al., 2016).

Since the P3b is evoked earlier than the prospective positivity, differences in the late time window between targets and deviants under the dual-task condition but not under the simple-task condition in our study can be explained by a later prospective positivity. Similarly, in Fowler (1994) where subjects had to control a virtual airplane and had to respond to targets with a motor response it could be discussed that expected P300 activity in their experiment was also superimposed by ERP activity related to PM tasks. Indeed they reported multiple peaks as well as slow waves evoked by task-relevant infrequent stimuli as an indication for overlaying ERP activity.

In summary, our findings can be interpreted well when assuming that under dual-task condition target stimuli evoke a P3b, which is sensitive to task emphasis, and at least one additional ERP component, i.e., a prospective positivity, is evoked. This requires the assumption that the dual-task condition in our experimental setup is also a PM task condition.

### Single-trial classification performance

Results of single-trial classification performance in case of using 62 channels show that infrequent stimuli (deviants or targets) can very well be distinguished from frequent task-irrelevant stimuli (standards) under both simple-task and dual-task condition. Further, our results show that task-relevance of target stimuli enhances differentiability under both task conditions for both time windows and different numbers of electrodes (see performance differences between target detection (t/st or t/d) and deviant detection (d/st) in Figure S3). This is even true for all investigated transfer cases. Thus, we could show that EEG activity evoked by targets can be detected in single trials with a higher performance. In particular, our results of single-trial classification performance support our findings of average ERP analysis (see Introduction question 1). Hence, findings of both average ERP analysis and single-trial classification support our assumption that motor-task relevance of stimuli affects the expression of parietal ERP activity as well as classification performance. These findings are also relevant for applications. It supports our earlier assumption (see Introduction question 2) that the capability of the user to recognize the task relevance of a stimulus has an effect on the ERP expression and classification performance of ERP activity evoked by task-relevant stimuli (see also Kirchner et al., 2013; Woehrle and Kirchner, 2014b,a; Kirchner et al., 2016).

The interesting finding that classification performance on both types of infrequent stimuli (targets and deviants) was significantly higher under simple-task compared to dual-task condition in case of using 62 channels (see Supplementary Materials in Figure S6) can be explained with the fact that overall more brain resources are assigned to the visual-motor oddball task under simple-task condition. This means that controlled conditions together with the usage of all possible data will not surprisingly result in best classification performance. Thus, looking at the differences in spatial distribution of the first retained xDAWN channel it can be seen that the xDAWN filter makes strong use of activity from parietal electrodes irrespective whether deviants or targets are classified from standards.

However, parietal activity is rather broad and a reduction of electrodes results in a decrease of information that can be used by the classifier and therefore results in an overall decrease in classification performance. It was found to be not sufficient to use 6 or less parietal electrodes to distinguish EEG activity evoked by infrequent task-irrelevant stimuli (deviants) from EEG activity evoked by frequent task-irrelevant stimuli (standards). In fact, deviant detection (d/st) was at chance level in case of using 6 or 2 electrodes. In contrast, target detection (t/st) was very high even in case of using only two electrodes. These findings support the assumption that EEG activity at parietal sites evoked by deviants is more similar to EEG activity evoked by standards than parietal EEG activity evoked by targets compared to standards. Further, these findings are also supported by the case that training took place on infrequent task-irrelevant (deviant) and infrequent task-relevant (target) events. In this case, targets could still be distinguished from deviants (t/d) with a reasonable performance even in case of using only two electrodes. Overall all findings support our assumption that parietal activity is relevant for the classification of EEG activity evoked by task-relevant stimuli. This clearly can be expected from average ERP expression at parietal electrode sites (see Introduction question 3).

Furthermore, it is obvious from xDAWN results that relevant features can be extracted from central areas for deviants (especially under simple-task condition), which likely results from a stronger weight of more centrally located processes likely correlated with P3a (Squires et al., 1975; Duncan-Johnson and Donchin, 1977; Verleger et al., 1994; Polich, 2007). Hence, parietal activity contributes less to the classification performance in case of the processing of deviant stimuli compared to target stimuli. This again is supported by our findings from average ERP activity that clearly show a less prominent expression of positive parietal ERP activity evoked by deviants compared to targets under both task conditions in both time windows.

When using two selected parietal electrodes (Pz and CPz) which are expected to mainly record brain activity involved in target recognition and PM processes, classification performance for target detection (t/st) in early window was higher under dual-task condition (which is assumed to involve target recognition *and* PM processes) than under simple-task condition (which is assumed to *not* to involve PM processes but only processes related to target recognition). Moreover, performance in target recognition (t/st and t/d) is similar between both task conditions in case that 6 parietal electrodes are used. Hence, controlled simple-task condition does no longer result in higher classification performance as discussed for the case that all 62 electrodes covering the whole head are chosen. This finding again supports that processes such as target recognition, task evaluation *and* PM processes contribute strongly to the classification of *target* events under dual-task condition (especially in case that parietal electrodes are chosen). This finding is supported by the finding that classifier transfer between task conditions for the distinction of targets from deviants (t/d) is strongly reduced in classification performance (see Figure 9), supporting a big difference between activity evoked by targets under simple-task compared to dual-task condition. Hence, especially ERP activity evoked by the PM related processes under dual-task condition is expected to contribute to the increase in classification performance under dual-task compared to simple-task condition in case that available data is limited to two parietal electrodes (CPz/Pz). This again mirrors findings from average ERP activity analysis.

Next, we want to address the issue of the effect of classifier transfer on the interpretability of results of single-trial classification performance (see Introduction question 4). While we found a very strong and clear mapping between classification performance and the expression of ERP components that are expected to be most typical for the differences in experimental design, results conducted after classifier transfer are likely more strongly influenced by the separability of the training data *per se* (see Ke et al., 2016 for discussion). Main differences between task conditions are lost due to classifier transfer. This is supported by the finding that classification performance is strongly increased for both windows in case that training took place on data from the dual-task scenario to distinguish between targets and standards in the simple-task scenario when only two electrodes are used (see Figure 9).

In the labyrinth oddball scenario investigated here, a transfer of classifier from dual-task condition to simple-task condition increases the separability of EEG trials evoked by targets compared to EEG trials evoked by standards in case that a very limited amount of training data (i.e., few relevant electrodes) is used. Especially, classification performance in distinguishing between targets and standards, i.e., target detection (t/st), is significantly higher in the transfer case (dual to simple) than in the no-transfer case (i.e., under simple-task condition) in both time windows (see Figure 9 and Supplementary Materials in Figure S6). However, in the reverse transfer direction (simple to dual), target detection (t/st) was significantly lower in the transfer case (simple to dual) than in the no-transfer case (i.e., under dual-task condition) for the late time window. Under dual-task condition the broader parietal ERP complex suggests higher variability in single trials which helps to train a robust classifier. This robust classifier improves classification performance in the transfer case (see similar findings in Kim and Kirchner, 2016).

In future work, the question of relevance of our results for rehabilitation robotics (see Introduction question 5) must be further evaluated. It must be tested whether our findings can be transferred into an application scenario to better answer the question whether a subject recognized task relevance of stimuli or not. This can be relevant for rehabilitation applications to answer the question whether task-relevant stimuli, e.g., commands of a serious game or from a therapist are recognized by the patient as task relevant. A similar transfer was shown in our earlier studies where the study goal was to adapt human-machine-interaction regarding the user's capability to bring attention to task relevant targets (Kirchner et al., 2013; Woehrle and Kirchner, 2014a,b; Kirchner et al., 2016). In our previous studies, training took place on standards and targets to detect unrecognized target events (i.e., missed targets) that were expected to be similar in ERP activity evoked by standards. Our results found in the presented study suggests the alternative approach, i.e., to train on targets and deviants for the differentiation of EEG activity evoked by the recognition or failure in recognition of task-relevance of target events (targets vs. missed targets) in applications. However, our results in the presented study do not answer the question whether this approach is outperforming the approach of training on standards and targets to detect trials (events) in which the patient recognized task relevance. Thus, classification performance achievable under both classification conditions (targets vs. standard or targets vs. deviants) to detect whether a patient understood task relevance of stimuli must still be evaluated under real application conditions. On the other hand, from our results it is quite clear that a transfer from a classifier trained in a simple setup (e.g., simple-task condition) to predict task recognition under multi-task condition (e.g., dual-task condition or an application which is very likely a multi-class condition) with the goal to infer whether a target was recognized by a patient as a task-relevant stimulus, is not a very feasible approach, since it would result in a strong decrease in classification performance. This decrease in performance can be explained by the big differences in ERP activity evoked by targets under dual-(motor-)task condition compared to simple-task condition. Hence, such an approach, as for example applied in Kim and Kirchner (2013, 2016) and Kim et al. (2017), is not suitable when using targets and deviants for training.

From the perspective of neuroscience methods (see Introduction question 5) the comparison of controlled experimental conditions with more natural complex conditions leads to interesting results, explainable by and supporting other work such as for example the work of West and colleagues who showed simultaneous occurrence of P3 and prospective positivity under certain conditions (West et al., 2003). Adding a second motor task (dual-task condition) will evoke additional parietal ERP activity although all stimuli are kept the same as under simple-task condition. Hence, more complex scenarios show that many processes usually run in parallel interacting with each other and are overlaid resulting in overlaying ERPs or ERP components as well as shifts in spatial expression (i.e., more parietal expressed P300 components evoked by deviants under dual-task condition than expected for P3a Polich, 2007). In future, it will be of interest to investigate the exact conditions under which different types of stimuli and human activity are interacting and to what extent. This research will expand our knowledge on the effects of different attributes of stimuli that go beyond attributes such as modality (Parasuraman and Davies, 1984; Wickens, 2008) for sharing attentional and cognitive resources. This understanding is not only relevant for a deeper understanding of brain processes but it will also help to design better human-machine-interfaces as shown already by, e.g., Kirchner et al. (2016).

In summary, obtained differences in performance of single-trial classification can be explained well by underlaying differences in ERP activity. However, local differences in ERP activity can be covered since the classifier is making use of more spatially distributed activity. The sensitivity of classification performance caused by more local differences in ERP activity can be enhanced by electrode selection. Depending on the selected electrodes, it cannot only be inferred from classification performance whether a subject just recognized an infrequent stimulus (deviant or target) and whether she/he will respond to such an infrequent stimulus but also whether such a response behavior infers with another task and requires changes in the task set or task switch (as in case of dual task performance). However, results clearly show that for the later question an extended set of electrodes is needed. Whether a set of electrodes covering the whole head (here 62 electrodes in an extended 10-20-system) or a smaller set of electrodes (>6) is sufficient must be investigated in future work. In case of classifier transfer which can be applied to improve classification performance one must, however, be careful when interpreting the results with respect to differences in brain processes, since class differentiability during training might have a stronger effect on classification performance than underlying differences in ERP activity. Most interestingly, classifier transfer can improve classification performance or reduce classification performance as it can be predicted by involved ERP activity.

## Conclusions

Results of this study are highly relevant with respect to the usability of single-trial classification performance as a tool for the investigation of brain processes. We showed that differences in single-trial classification performance can well be explained by the strength of expression in average ERP components and the other way around. Further, different data selections by reducing available electrodes results in changes in classification performance that are expected given the knowledge on involved brain activity. However, single-trial classification performance is very sensitive regarding general separability of classes especially in case that available data is reduced by e.g., electrode selection. Thus, while we found strong correlations between classification performance and expression of ERPs that are expected to contribute to class differences, after classifier transfer results were also influenced by general class separability in training data in case of electrode reduction. While we showed that single-trial classification performance has a good chance to become a strong tool for the interpretation of brain processes, especially with respect to questions of how strong specific activities influence the whole processing in the brain, further investigations are needed to develop policies that guide the user for a systematical use. Only following such a guide allows the use of single-trial classification performance as well as classifier transfer approaches as tools to understand brain processes. By investigating brain activity by different methods, e.g., by ERP average analysis as well as by machine learning, results can be evidenced. Hence, additional methods that allow for an interpretable analysis of brain processes are highly relevant and should be considered for the analysis of brain processes.

Besides the strong influence of BCIs as a paradigm shift in neuroscience showing that single-trial analysis is an appropriate method to investigate brain activity, recent researches in BCIs showed that investigating brain activity under natural condition instead of lab situation is feasible. This led to new approaches in clinical applications such as rehabilitation making use of online single-trial EEG analysis to close the loop between the brain and the body. Here we showed that during complex visual-motor activity it is possible to detect ERP activity related with target recognition and task-change processes. This can be used to improve the movement prediction when patients are instructed to execute specific movements by a therapist or serious game (Wöhrle et al., 2017). Our results support that for strong activities such as P300 and related processes single-trial ERP detection is in principle making use of features that are physiologically interpretable and expected. However, we showed that a selection of electrodes based on knowledge on the brain processes can even enhance interpretability. This might be of interest in case that a specific brain process should be detected. While the P300 complex is a very strong and prominent activity, even for this ERP complex a reduction of electrodes is improving interpretability, however, with the cost of classification performance. Hence, while we showed that target-related activity can be well detected in application with multi-motor activity, a reduction of electrodes will lead to a reduction of classification performance and must thus be further evaluated especially with respect to applicability. Classifier transfer might be a good approach to improve classification performance.

## Author contributions

EK designed and set up the experiment and analyzed the ERP data. EK and SK recorded data. SK performed machine learning analysis. EK wrote introduction, discussion and conclusions. EK and SK wrote methods and results sections. SK and EK designed statistical analysis. SK performed statistical analysis. SK created most figures in this manuscript.

### Conflict of interest statement

The authors declare that the research was conducted in the absence of any commercial or financial relationships that could be construed as a potential conflict of interest.
