# Supplementary material for: Multi-Tasking and Choice of Training Data Influencing Parietal ERP Expression and Single-Trial Detection—Relevance for Neuroscience and Clinical Applications
Source: Front Neurosci. 2018 Mar 27;12:188. doi: 10.3389/fnins.2018.00188 (PMC5881241; doi:10.3389/fnins.2018.00188)
Supplement: Supplementary file 1 [file DataSheet1.pdf]

Supplementary Material: Multi-tasking and Choice of Training Data Influencing Parietal ERP Expression and Single-trial Detection - Relevance for Neuroscience and Clinical Applications

Elsa Andrea Kirchner* and Su Kyoung Kim
*Correspondence:
Author Name: Elsa Andrea Kirchner
ekir@informatik.uni-bremen.de

1 SUPPLEMENTARY TEXT

1.1 Average ERPs of individual subject for both task conditions

Average ERPs of individual subjects for both task conditions (dual and simple task) are depicted in Figure S1. Detailed information about procedures of ERP analysis is reported in main text.

1.2 Statistical results of ERP analysis (averaged ERP activity)

ERP amplitude values across subjects were analyzed by repeated measures ANOVA with four within-subjects factors: stimulus type (standard/deviant/target), window (early/late), electrode (CPz/Pz/POz), and task condition (simple/dual). Detailed information about statistical analysis and results are reported in the main text. Here, relevant parts of statistical results are illustrated in Figure S2. Figure S2-A shows the comparison between stimulus types (standard/deviant/target). Figure S2-B shows the comparison between task conditions (simple/dual) in ERP activity.

1.3 Statistical results of machine learning analysis (single-trial ERP detection)

Binary classification performances were analyzed by repeated measures ANOVA with five within-subjects factors: stimulus type, window type, number of channels, task condition, and transfer condition. Here, all statistical results are illustrated in Figure 3–6. Figure S3 shows the comparison between stimulus types: standards vs. targets, standards vs. deviants, and deviants vs. targets. Figure S4 shows the comparison between channel types: 62 channels, 6 parietal (CP3, CP4, CPz, P3, P4, Pz) and 2 parietal (CPz, Pz) channels. Figure S5 shows the comparison between task conditions and transfer types. Figure S6 shows the comparison between windows.

1.4 Investigation of effect of response time on positive parietal ERP complex

Earlier preliminary results suggest that a delay in response affects the classification performance as well as expression of the positive parietal ERP complex (Kim and Kirchner, 2012). These results have to be considered as preliminary, since only two subjects were investigated. In short, 6 EEG data sets (each 120 targets and 720 standard stimuli) were collected from two subjects during multi-tasking in a virtual environment. EEG was recorded with a 64 electrode actiCap system and BrainAmp DC amplifiers (Brain

Products GmbH) with reference at FCz. The subjects played a physical simulation of the BRIO® labyrinth and had to respond to infrequent *target* stimuli by pressing a buzzer. Deviant (infrequent non-target) stimuli were not presented to the subjects. The subjects were instructed to wait before responding to *target* stimuli until they had guided the ball into a safe corner. The 6 data sets of two subjects were merged into one data set for each subject. To find how the task reaction time influences the classification performance, the data was divided into 5 groups based on reaction time (RT in ms): RT 1400, RT 1600, RT 1800, RT 2000, and RT 7000.

The data for each group was resampled 10 times using bootstrapping technique by randomly choosing 60 training examples. To avoid the influence of training size, the number of training examples for *target class* (n = 60) was kept constant for each RT group. The remaining examples were used to test the classifier. Finally, we obtained 20 classification values for each RT group (10 values x 2 subjects for each RT group). As a performance metric, we used balanced accuracy (bACC).

To find an effect of task reaction time on the single-trial ERP detection, the data was analyzed by repeated measures ANOVA with reaction time (RT) as a within-subjects factor (5 levels: RT 1400, RT 1600, RT 1800, RT 2000, RT 7000). For pairwise comparisons, Bonferroni correction was applied. The observed ERP amplitude difference (Fig. S8), i.e., average ERP across two subjects between *targets* and *standards* was not statistically analyzed due to small sample sizes.

The classification performance of the positive parietal ERP complex was reduced by the delay of the response (see Fig. S7).The more the task response time was delayed, the more the classification performance of the positive parietal ERP complex was reduced. The intensity of the positive parietal ERP complex was reduced by the delay of the response (see Fig. S8). The more the task response time was delayed, the more the amplitude of the positive parietal ERP complex was reduced.

REFERENCES

Kim, S. K. and Kirchner, E. A. (2012). Preliminary results on p300 detection using machine learning when modulating task reaction time. In Proceedings of the 18th Annual Meeting of the Organization for Human Brain Mapping, OHBM-2012, Beijing, June 10-14.

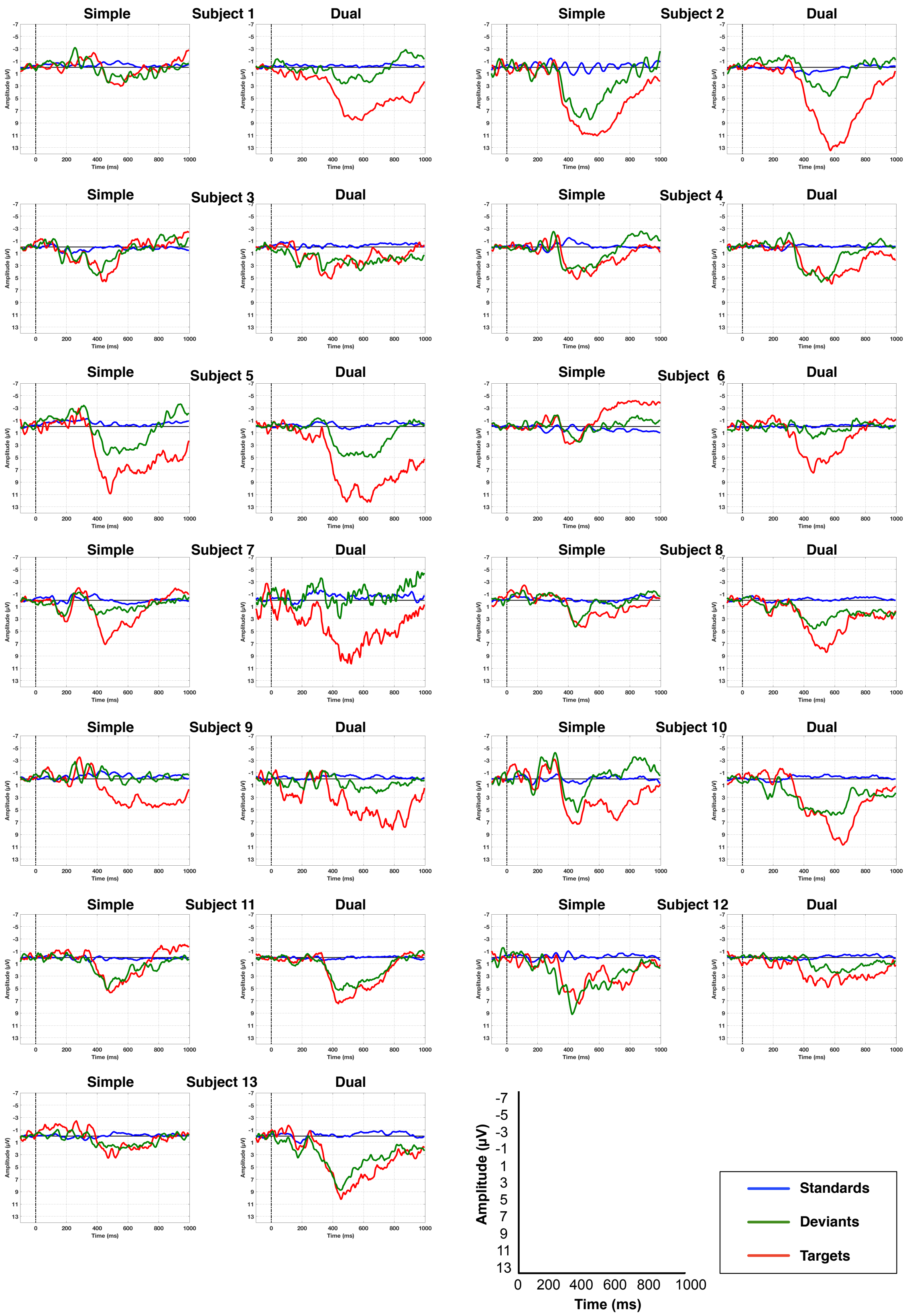

Figure S1. Average ERP of individual subjects for both task conditions (simple and dual task).

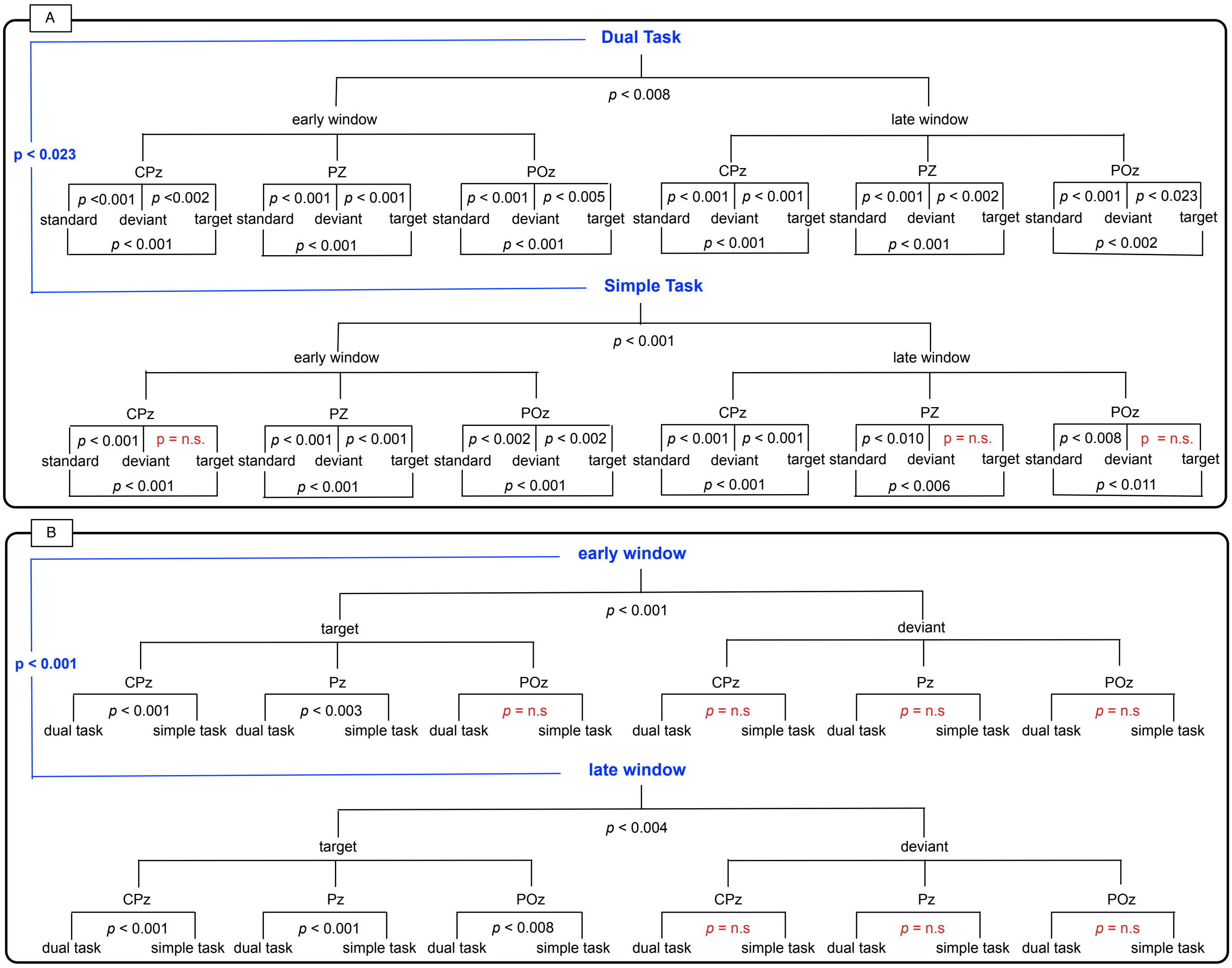

Figure S2. Statistical results on average ERP activity: comparison between stimulus types (A) and comparison between task conditions (B). Insignificant differences are marked in red.

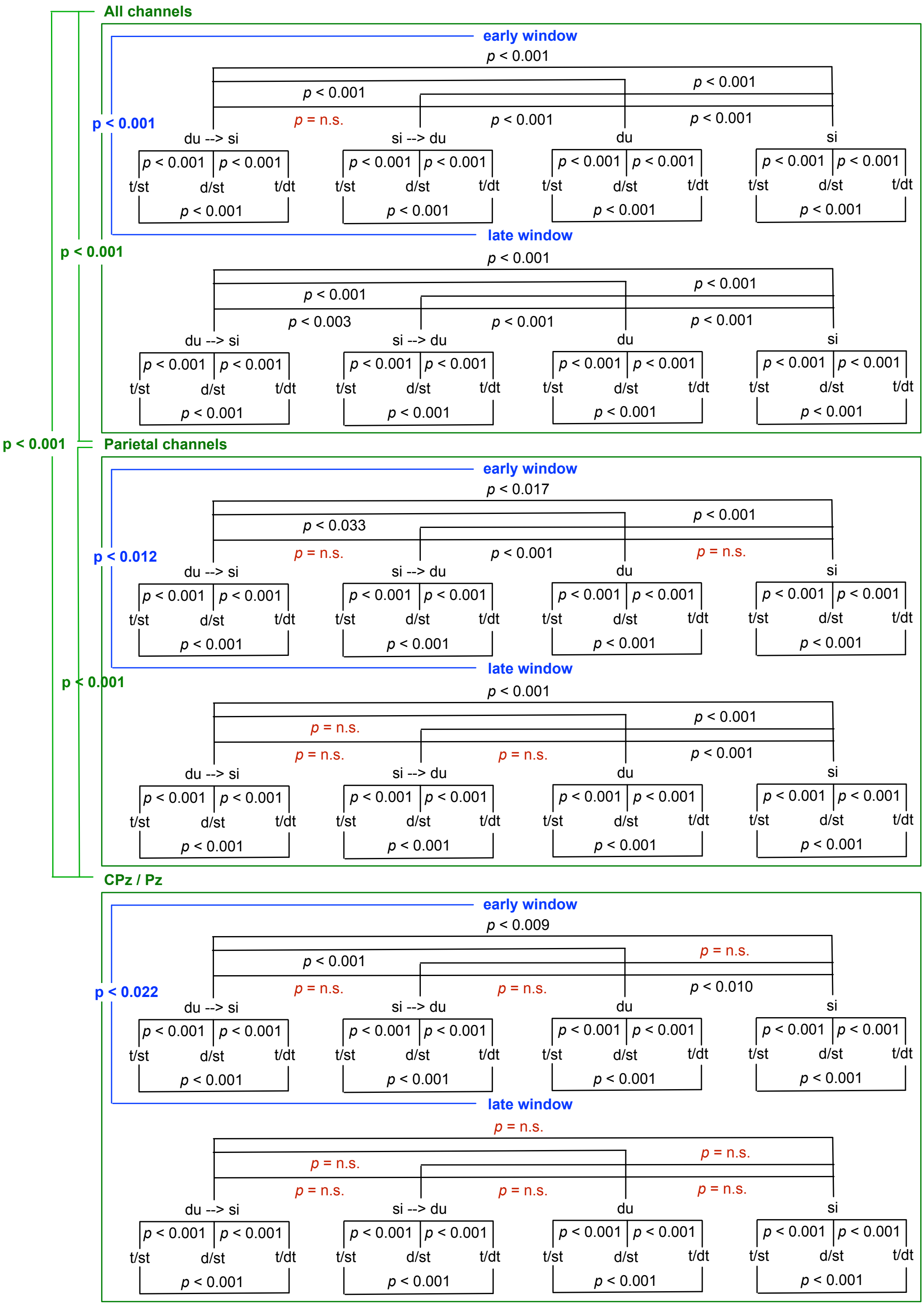

Figure S3. Statistical results on single-trial detection: comparison between three types of ERP detection: target detection (t/st: targets vs. standards), deviant detection (d/st: deviants vs. standards), target detection (t/d: targets vs. deviants). Insignificant differences are marked in red.

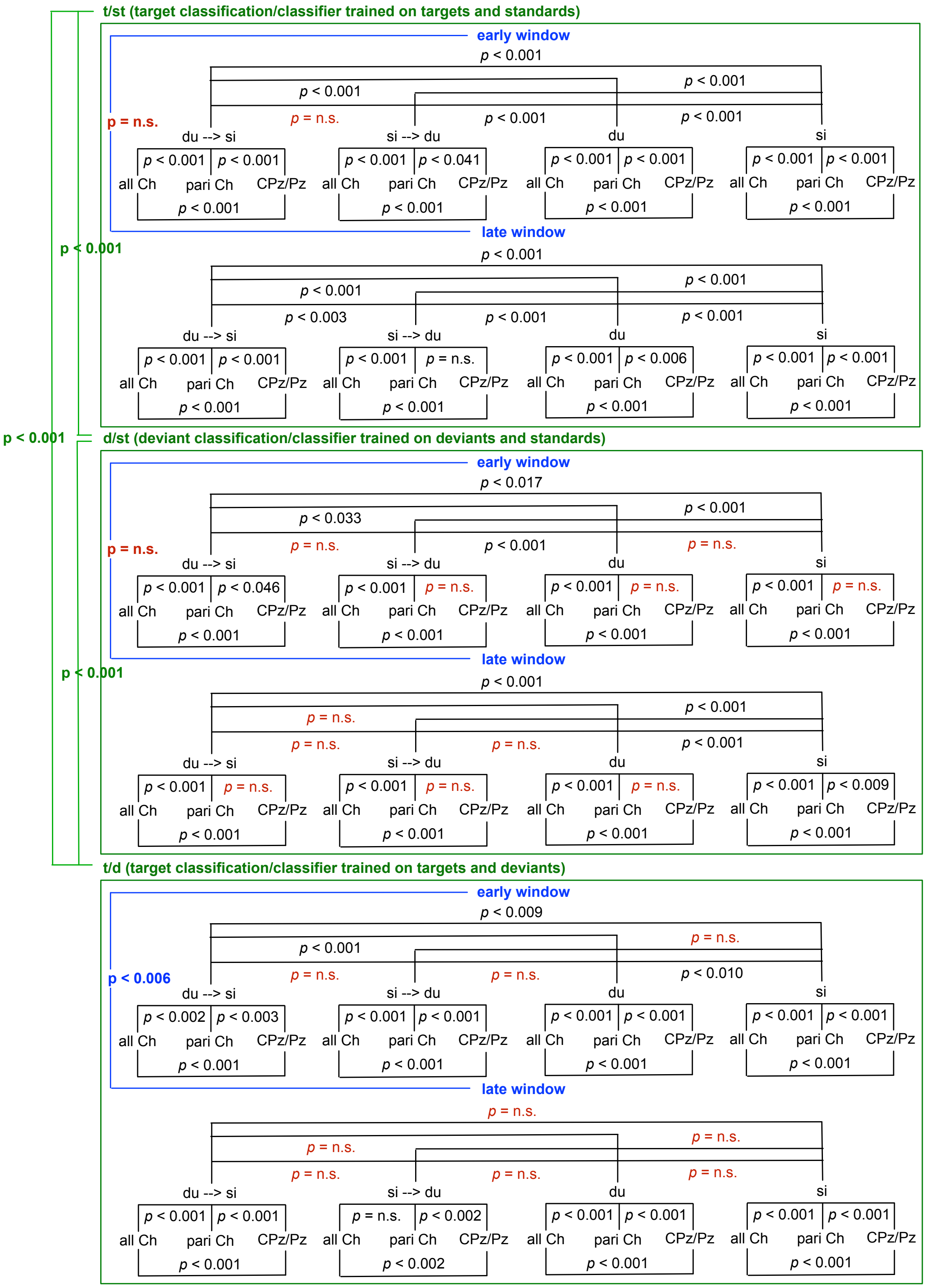

Figure S4. Statistical results on single trial detection: comparison between channel types (62 channels, 6 and 2 parietal channels). Insignificant differences are marked in red.

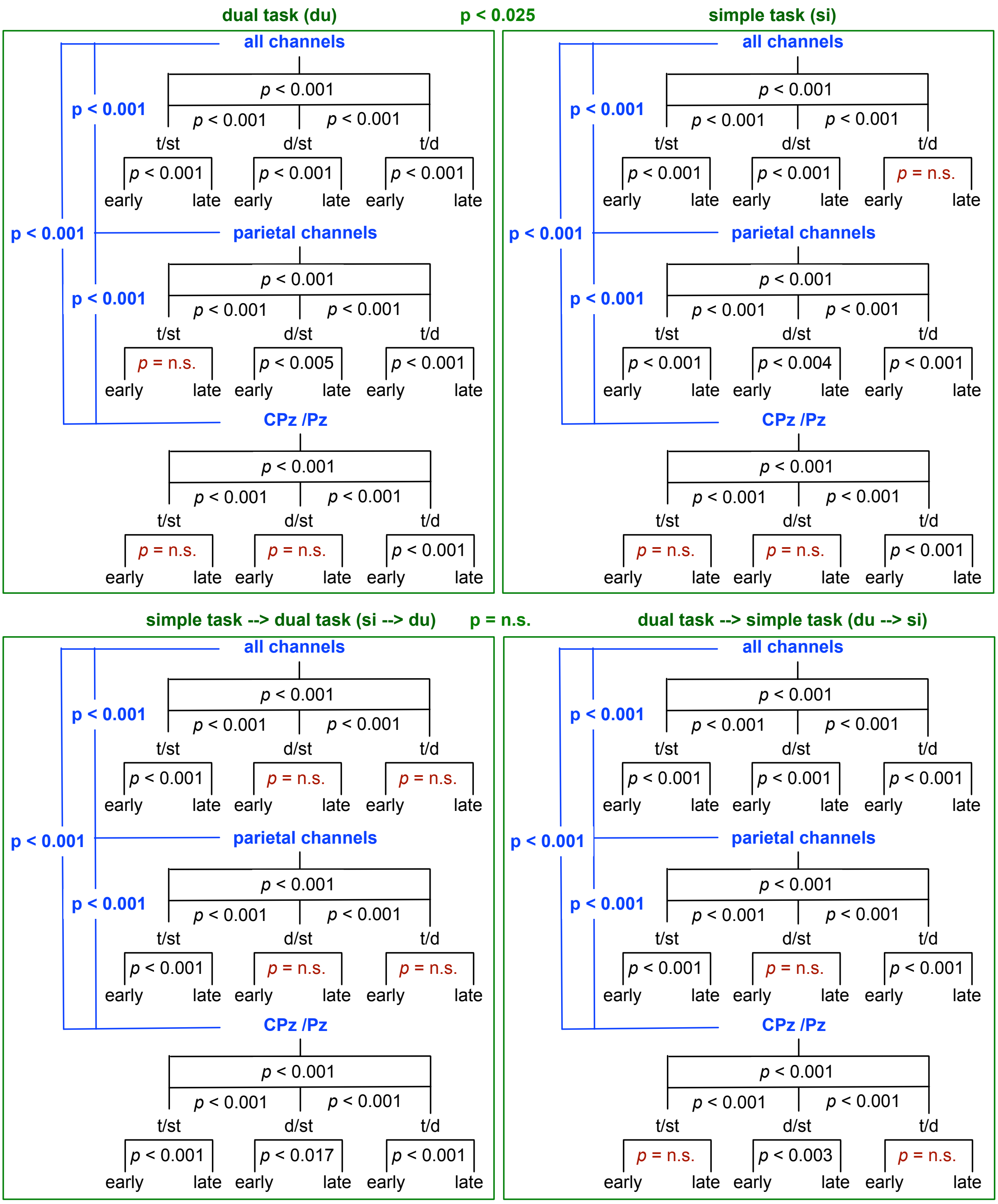

Figure S5. Statistical results on single-trial detection: comparison between task types (simple and dual task) and transfer types (transfer case and no transfer case). Insignificant differences are marked in red.

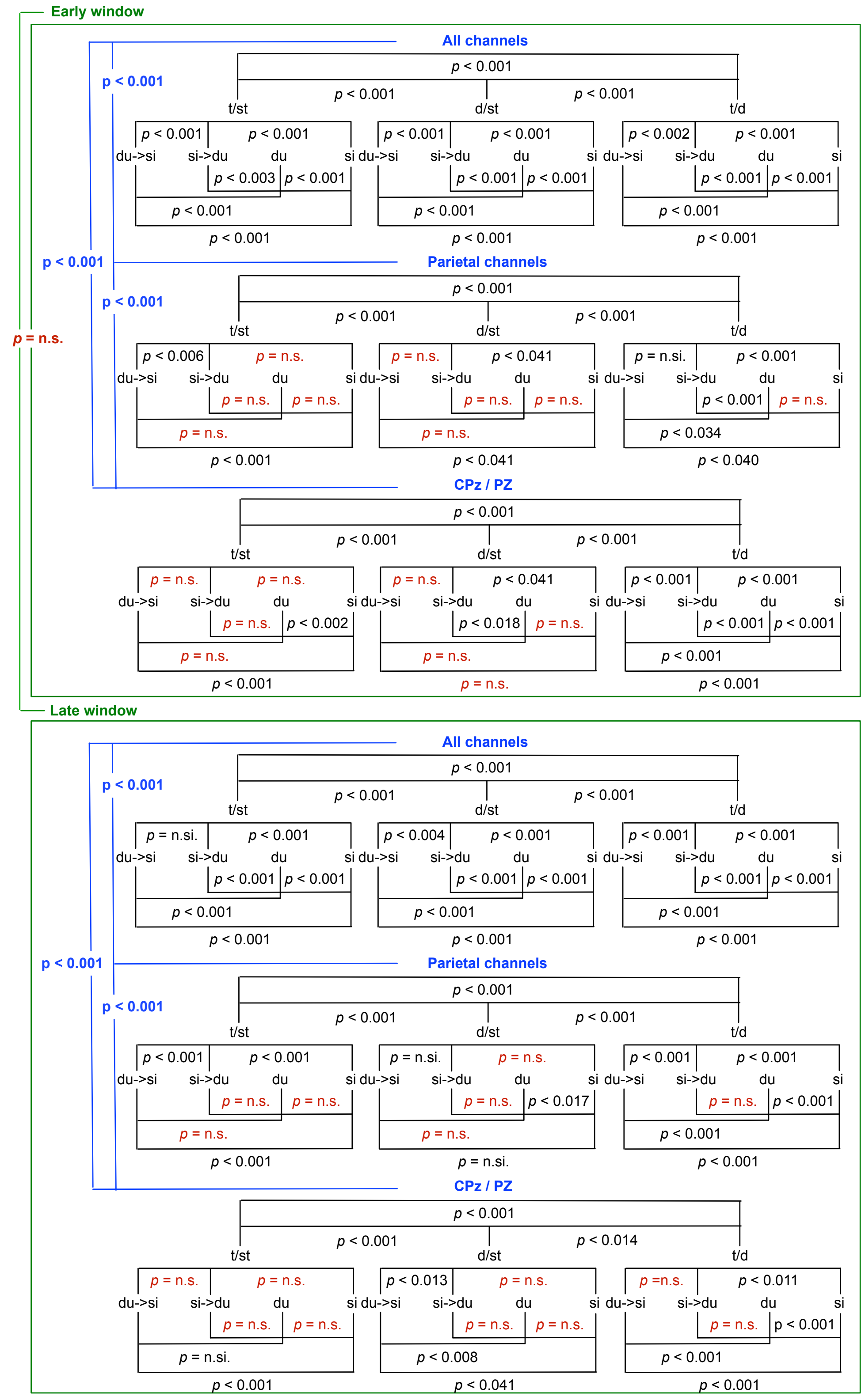

Figure S6. Statistical results on single-trial detection: comparison between window types (early and late window). Insignificant differences are marked in red. Further statistical values between task conditions and transfer conditions across three types of channels, both window types, and three different ERP detection types are reported: du vs. si → du: $p < 0.001$, du vs. du → si: $p < 0.002$, si vs. du → si: $p < 0.001$, si vs. si → du: $p < 0.001$.

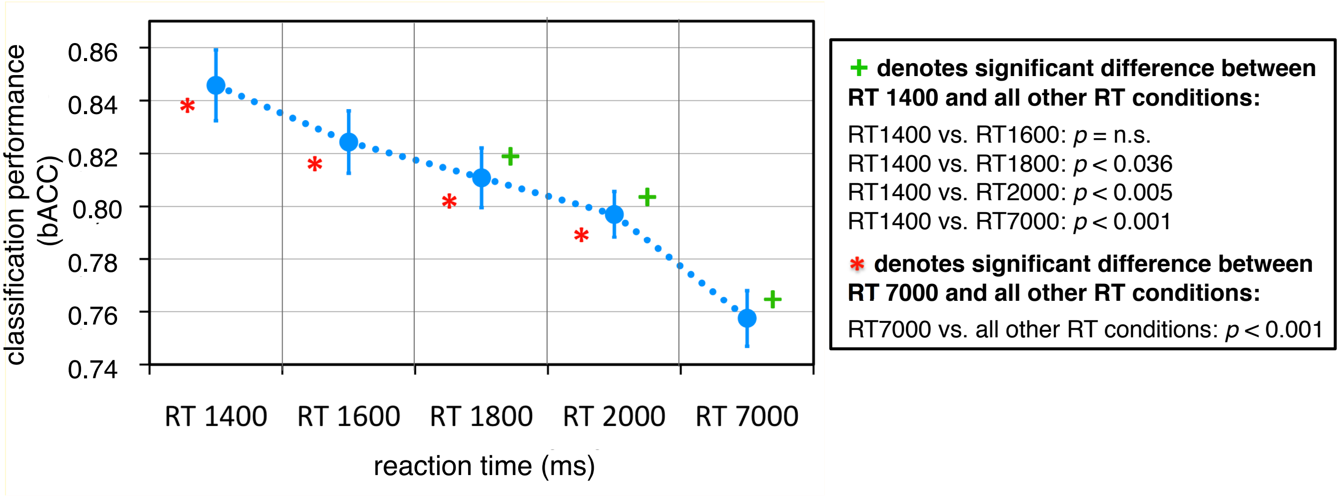

Figure S7. Classification performance in the detection of the positive parietal ERP complex using machine learning. The mean of 20 balanced accuracy (bACC) for each RT group and standard error of mean is depicted. The classification performance of the positive parietal ERP complex was reduced by the delay of the response. The more the task reaction time was delayed, the smaller the classification performance of the positive parietal ERP complex became. Figure is lightly changed from Kim and Kirchner (2012).

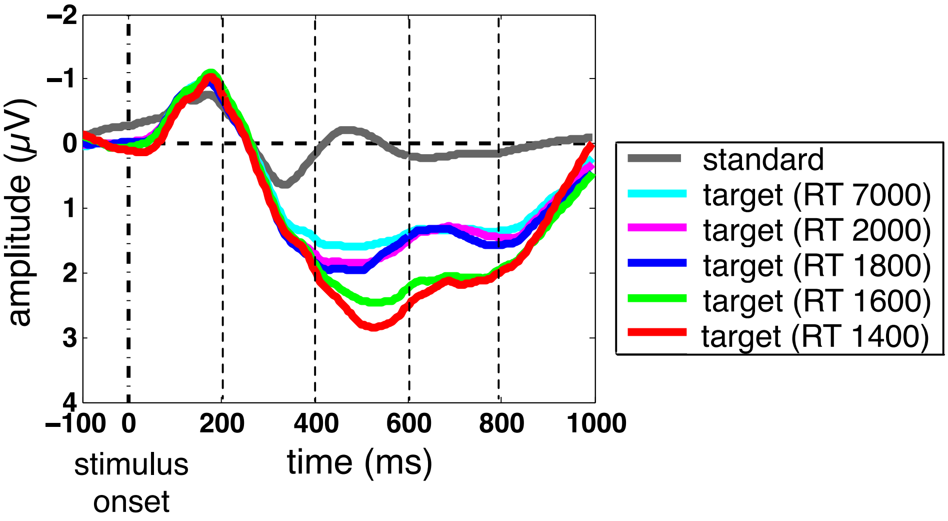

Figure S8. Average ERPs elicited by *targets* (colored lines) and *standards* (grey line) at electrode Pz (RT: reaction time). The intensity of the positive parietal ERP complex was reduced by the delay of the response. The more the task reaction time was delayed, the less the intensity of positive parietal ERP complex. Figure is lightly changed from Kim and Kirchner (2012).
